# Model systems to study *Mycobacterium tuberculosis* infections: an overview of scientific potential and impediments

**DOI:** 10.3389/fcimb.2025.1572547

**Published:** 2025-05-08

**Authors:** Prachi Nangpal, Neha Lalwani Nagpal, Nupur Angrish, Garima Khare

**Affiliations:** Department of Biochemistry, University of Delhi, New Delhi, India

**Keywords:** tuberculosis, animal models, omics-based approaches, host-pathogen interactions, cellular models

## Abstract

Despite years of global efforts to combat tuberculosis (TB), *Mycobacterium tuberculosis* (*Mtb*), the causative agent of this disease, continues to haunt the humankind making TB elimination a distant task. To comprehend the pathogenic nuances of this organism, various *in vitro, ex vivo* and *in vivo* experimental models have been employed by researchers. This review focuses on the salient features as well as pros and cons of various model systems employed for TB research. *In vitro* and *ex vivo* macrophage infection models have been extensively used for studying *Mtb* physiology. Animal models have provided us with great wealth of information and have immensely contributed to the understanding of TB pathogenesis and host responses during infection. Additionally, they have been used for evaluation of anti-mycobacterial drug therapy as well as for determining the efficacy of potential vaccine candidates. Advancements in various ‘omics’ based approaches have enhanced our understanding about the host-pathogen interface. Although animal models have been the cornerstone to TB research, none of them is ideal that gives us a complete picture of human infection, disease and progression. Further, the review also discusses about the newer systems including three dimensional (3D)-tissue models, lung-on-chip infection model, *in vitro* TB granuloma model and their limitations for studying TB. Thus, converging information gained from various *in vitro* and *ex vivo* models in tandem with *in vivo* experiments will ultimately bridge the gap that exists in understanding human TB.

## 1 Introduction

Tuberculosis (TB) remains a leading cause of death by a single infectious agent, claiming an estimated 1.09 million deaths among HIV-negative people and an estimated 161,000 deaths among HIV-positive people in the year 2023 (WHO Global TB report, 2024). A total of 1.25 million deaths in 2023 was observed, which was lower than the number of deaths observed in the year 2019. However, this reduction in total number of TB deaths was calculated to be 23% between 2015 and 2023, which is far from the desired milestone of the WHO End TB Strategy (a 75% reduction between 2015 and 2025) (WHO End Strategy). Unawareness among the common people is one of the main causes for non-compliance and non-adherence observed to the TB treatment, which often leads to emergence of multidrug and extensively drug-resistant strains. In 2023, 400,000 individuals developed MDR/RR-TB worldwide, with 150,000 deaths due to multidrug-resistant TB. Thus, elimination of TB is an urgent matter and requires global efforts to attain the targets of 90% reduction in TB incidence, with fewer than 100 cases per million people by 2035, as per the WHO End TB Strategy (WHO End Strategy). Achieving these targets demand the requirement of developing novel and effective TB vaccines, improved diagnostic measures and better therapeutics.

**Figure 1:**
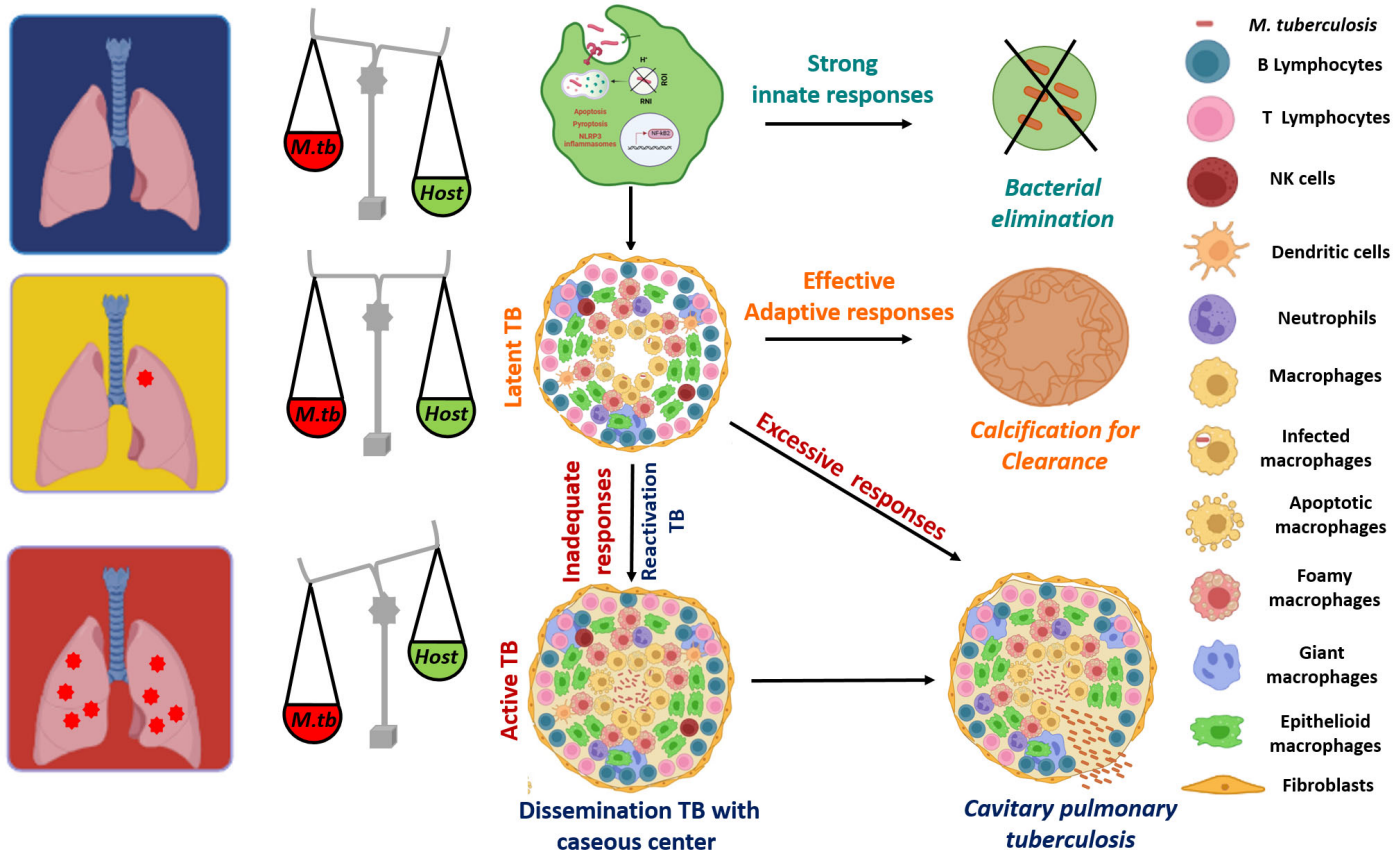
A wide spectrum of clinical fates of *Mtb* infection: Alveolar macrophages are the first cells to get exposed to *Mtb* and hence are the most important line of defense that decides the fate of the infection. Many individuals do not get infected with *Mtb*, due to the robust innate immunity that leads to an efficient activation of macrophages, resulting in a stronger anti-mycobacterial response and elimination of the pathogen. However, most of the individuals who get infected with *Mtb* enter into latency (Latent TB) and an effective adaptive response is able to control the infection in a few intact granulomas consisting of various immune cells that act as safeguards to contain the infection. With a successful adaptive response, some latent individuals also sterilize the infection via calcification. Some 5-10% of infected individuals develop active TB with cavitary pulmonary tuberculosis becoming a potential source of transmitting the pathogen to other uninfected individuals. In addition, ~ 90% of the active TB cases are results of reactivation of latent infections, due to the immune comprised status (HIV infection, anti-TNF-α therapies) of these individuals and thus, with inadequate immune responses, granulomas become necrotic with a caseous center (soft, cheese-like appearance), which results in dissemination of the infection to other parts of the host system. The figure is prepared by using BioRender.com.

*Mycobacterium tuberculosis* (*Mtb*) is an extremely successful pathogen that has daunted the mankind since ages. The wide clinical outcomes that are experienced on *Mtb* infection complicate our understanding on the host-pathogen interface as well as the immune responses required to eliminate the pathogen. Few individuals do not get infected at all despite being exposed to *Mtb* reflecting the importance of strong innate immune defenses (**Figure 1**) (Stead et al., 1990; van Crevel et al., 2002; Simmons et al., 2018; Kroon et al., 2020; Boom et al., 2021; Aiello et al., 2023). However, most of the individuals who get infected with *Mtb*, enter into latency (LTBI) and control the infection in a few intact granulomas. These individuals are tuberculin skin test (TST) positive and about 5-10% of these individuals are at risk of developing active TB during their lifetime (van Crevel et al., 2002; Bucsan et al., 2019). Besides, certain other underlying health conditions including diabetes, HIV infection or immunotherapies pose a significant risk of reactivation TB in LTBI individuals (Miller and Ernst, 2009; Kwan and Ernst, 2011; Ai et al., 2016; Bucsan et al., 2019; Tezera et al., 2020a; Salindri et al., 2021). 5-10% of the individuals who get infected with *Mtb* develop the active full-borne disease and are potential sources of transmitting the pathogen to the uninfected individual (Acharya et al., 2020).

*Mtb* is transmitted to the uninfected host via the aerosols propelled by the patient’s cough. The bacilli reaches the alveoli where it infects the alveolar macrophages, the first cells to get exposed to the pathogen. Recognition of *Mtb* by various PRRs (pattern recognition receptors) leads to phagocytosis of pathogen and induction of a plethora of innate responses (Liu et al., 2017; Chai et al., 2020; Boom et al., 2021; Chandra et al., 2022). Subsequently, macrophage activation stimulates the anti-mycobacterial defenses including acidification of the infected phagosomes, ROI and RNI stress, apoptosis and production of proinflammatory cytokines (Liu et al., 2017; Boom et al., 2021). However, being a smart pathogen, *Mtb* has premeditated its machinery to evade the host antibacterial artillery and finally persist inside macrophages as an intracellular pathogen. Various *Mtb* proteins are known to inhibit the assembly as well as the acquisition of the NADPH oxidase on the infected phagosomes, thereby reducing the generation of reactive oxygen species (Sun et al., 2013; Köster et al., 2017; Srivastava et al., 2019). It has also devised its own detoxification system based on *katG* to quench the oxidative radicals (Ng et al., 2004). In addition, *Mtb* expresses proteins that prevent the fusion of phagosomes to lysosomes, thus, stalling the delivery of the bacteria to the lysosomes (Puri et al., 2013; Pradhan et al., 2018). *Mtb* also encodes proteins that help the pathogen survive the acidic conditions faced inside the phagosomes (Vandal et al., 2009; Singh et al., 2019). Besides, virulent *Mtb* induces necrosis of infected macrophages in order to enhance its spread to other macrophages to ultimately increase bacterial replication (Divangahi et al., 2013). The pathogen induced dysregulation of the antibacterial responses mark the rapid division of *Mtb* until the adaptive immune responses come into the picture.

Following the macrophage infection, other innate cells such as neutrophils, dendritic cells and natural killer cells also traffic to the site of infection in an attempt to effectively control the pathogen (Ravesloot-Chávez et al., 2021; Sankar and Mishra, 2023). Dendritic cells are key players in disseminating the bacteria to the draining lymph node where they present *Mtb* antigens to prime the T cells (Marino et al., 2004). Following priming, an adaptive immune response is mounted, which includes the involvement of various immune cells like CD4+, CD8+ T and B-cells. Moreover, CD4+ T cell responses play a major role in anti-TB immunity, as depletion of these cells such as in the case of HIV infection, renders an individual more susceptible to tuberculosis. These CD4+ T cells differentiate further leading to the generation of effector and memory T cells. Trafficking of these effector T cells and other immune cells to the lungs initiate the cell-mediated responses that ultimately lead to granuloma formation (Chandra et al., 2022). Granulomas are pivotal to TB immunity, which are well-organized and structured complex, comprising of collection of various immune cells such as macrophages, neutrophils and lymphocytes surrounded by fibrinogen and collagen (Chai et al., 2020). As the infection progresses, macrophages within the granulomas mature to differentiate into various phenotypes including differentiated or epithelioid macrophages, foamy macrophages, and multinucleated (or Langhans) giant cells (MNGCs) (Ndlovu and Marakalala, 2016; Chai et al., 2020). A successful adaptive response will either control the infection within these stable granulomas (latent TB) or sometimes even sterilize the infection leading to sclerotic and calcified granulomas (Chandra et al., 2022). In latent infections, granulomas halt the bacterial replication and spread, however, are unable to eliminate the pathogen (Luies and Du Preez, 2020; Alsayed and Gunosewoyo, 2023). Thus, *Mtb* can survive in this conducive niche in a non-replicating state for decades (Luies and Du Preez, 2020; Alsayed and Gunosewoyo, 2023). These individuals are at a lifetime risk of reactivation, which is estimated to be around 5-10%, with most of the reactivation TB cases observed to be within the first five years of initial infection (WHO; Martin et al., 2021). Infact, ~90% of the active TB cases are due to reactivation of latent infections (Jilani et al., 2020; Martin et al., 2021). In individuals with inadequate adaptive response in case of HIV infections, newborns or people undertaking anti-TNF-α therapies, granulomas become necrotic with a caseous center (soft, cheese-like appearance), which results in dissemination of the infection to other parts of the host (Solovic et al., 2010; Tornheim and Dooley, 2017; Chandra et al., 2022). Moreover, an excessive immune response can be detrimental rather than beneficial for the host, leading to worsened pathology, which is associated with cavitary pulmonary disease (**Figure 1**) (Comstock et al., 1974; Kaufmann, 2003; Tezera et al., 2020b). Infact, it is observed that as granuloma matures, *Mtb* influences the differentiation of the macrophages to foamy phenotype characterized by the accumulation of lipid bodies (Singh et al., 2012; Agarwal et al., 2021). These lipid bodies, which are induced by the pathogen by modulating lipolysis of the neutral lipids serve as both a nutrient source and a privileged niche for its replication (Singh et al., 2012). Thus, these foamy macrophages contribute to both bacterial sustenance and tissue pathology, which ultimately leads to cavitation. Thus, an inadequate immune response or an excessive host response leads to different forms of active TB (Tezera et al., 2020b). Due to such wide variety of clinical presentations that can be experienced upon *Mtb* infections, we currently lack knowledge about the immunological determinants that can distinguish between protection and pathology (**Figure 1**). The lack of information on the protective responses that can lead to containment or elimination of the pathogen along with those that can be detrimental to the host is a significant roadblock to the development of novel preventive vaccines against TB.

Animal models have been instrumental in providing a platform to increase our repertoire of knowledge on immune mechanisms and pathology driving factors. Much of our learning about the bacterial as well as host traits involved in TB infection and progression have come from the experiments conducted in various animal models. Although several animal models for TB exist including mouse, guinea pigs, non-human primates (NHPs) and rabbits etc., unfortunately, none of them recapitulates the complete picture of the pathological features of human TB disease. In this review, an attempt has been made to describe the pros and cons of various animal models along with a comparison of the respective pathological features they demonstrate and their usage for various aspects of TB research. Further, advancements made in the “omics-based approaches” have also been extremely beneficial in giving us insights into various host-pathogen interactions that are useful in developing host-directed therapies, are also discussed in the review.

## 2 *In vivo* models for TB research

Human samples from various healthy individuals and TB patients (active, latent, household contacts) are definitely the preferred choice for gaining real insights into various stages of *Mtb* infection. However, the limitations associated with human samples are the availability issues, the invasive procedures required to obtain the biopsies and ethical regulations that confine their usage (Soldevilla et al., 2022). Experimental animal models have been paramount in TB research and have provided valuable information about the pathogenesis as well as TB immunity. Apart from advancing knowledge on basic sciences, they have also been extensively employed as powerful research tools for translational research, including vaccine and drug testing. For instance, mouse model of experimental TB has been employed for rapid screening of compounds for their anti-TB activity due to the ease of delivering drugs orally. Moreover, guinea pigs are considered suitable for vaccine testing because of the immunological similarities they share with human disease. Infact, NHPs display clinical symptoms as well as granuloma structures similar to a human TB patient, making them a pragmatic and rational choice for studying immunological parameters and mechanisms (Zhan et al., 2017).

### 2.1 Mouse

Till date, the mouse (*Mus musculus*) model has been the most extensively employed animal system, not only for TB research but also for studying many other communicable and non-communicable diseases, due to the huge practical and cost-effective advantage it brings along. Additionally, the major reasons for its broad usage are: (i) the huge genomic similarity it shares with human genome, (ii) tools for genetic manipulation and (iii) the availability of several genetic knockout mouse strains. These knockout strains have provided us useful information on identification of various genetic factors associated with TB susceptibility, various innate and adaptive immune mechanisms and host-pathogen interactions.

Cost definitely plays an important role in the wide utility of mice for conducting various kinds of research, especially in context to TB, where requirement of BSL3 facility adds additional expense. Besides, the ease of handling and genetically manipulating these animals have provided further advantage in recognizing the importance of host responses, particularly T cell immunity required for protection against TB infection. For instance, mice deficient in CD4+ T cells have impaired ability to control *Mtb* and succumb to the disease much faster than the wild type mice (Caruso et al., 1999). Additionally, mice that are unable to produce T-bet (T-box transcription factor essential to Th1-cell generation), which is associated with reduced production of IFN-γ and increased production of IL-10 and accumulation of multinucleated cells in the lungs, are susceptible to virulent *Mtb* infection (Sullivan et al., 2005). In addition, many other immune cells contributing towards host response to *Mtb* infection have been identified in the mouse model, including γδTCR T cells, NK cells and iNKT cells (Orme and Ordway, 2016).

Toll like receptors (TLRs) are family of pattern recognition receptors (PRRs) that are involved in the recognition of various pattern associated molecular patterns (PAMPs) on the invading pathogen and mounting the downstream inflammatory response. TLRs are very well studied in mice to understand their function in TB resistance. For example, TLR4-/- deficient C3H/HeJ mice showed higher bacterial burden, reduced macrophage recruitment, higher IL-10 levels and higher neutrophil counts, leading to excessive lung inflammation and reduced survival (Abel et al., 2002; Park et al., 2020). It was also demonstrated that TLR2 and TLR9 work in cooperation to provide protection against TB. A TLR2/9 double knockout mice showed increased susceptibility to *Mtb* infection, which was related to defective proinflammatory cytokine production and impaired IFN-γ recall responses, along with altered lung pathology (Bafica et al., 2005). These results from various mice studies have been extended in humans, as polymorphisms in these TLR genes result in enhanced susceptibility to TB in various populations (Ogus et al., 2004; Zaki et al., 2012; Torres-García et al., 2013; Jafari et al., 2016). Several other PRRs, including C-type lectin receptors and NOD like receptors have also been shown to be important for innate immunity, by using murine models (Divangahi et al., 2008; Wilson et al., 2015).

Other genetically modified mice have also been used to study various components of signaling cascades involved in host responses to TB. Most of the TLRs work via stimulation of the MyD88 downstream protein that leads to NFκB activation, resulting in production of proinflammatory cytokines. Thus, MyD88-/- deficient mice displayed markedly increased bacterial burden, exacerbated pulmonary inflammation and necrosis (Scanga et al., 2004). These mice also showed a reduced survival time of 42 days post *Mtb* challenge, in comparison to wild type infected mice, that showed a survival time of >180 days (Scanga et al., 2004). Much of our knowledge about various other immune components such as cytokines like IFN-γ, TNF-α, IL-12 etc., has been determined in mice, in part due to the availability of large number of immunological reagents and antibodies, standardized assays and use of flow cytometry technique. Low-dose *Mtb* infection of IFN-γ-/- deficient mice, either via aerosols or intravenously resulted in an increased number of acid fast bacilli along with heightened pathological damage and succumbed to disease faster (Cooper et al., 1993; Flynn et al., 1993). In yet another study, the use of murine model established the essentiality of TNF-α and its receptor in TB immunity (Flynn et al., 1995). Murine models have been used to establish the role of many other cytokines of innate and adaptive immunity, including IL-12, IL-17 and IL-23 (Cooper et al., 2002; Méndez-Samperio, 2010; Okamoto et al., 2010; Torrado and Cooper, 2010; Khader et al., 2011). Infact, it was observed that children with genetic defects in IL-12/23–IFN-γ axis showed Mendelian susceptibility to mycobacterial and other infectious diseases (Filipe-Santos et al., 2006). Further, the discovery of inhalation chambers marked a new era of TB research and enabled us to study *Mtb* infection cycle through its natural route of aerosol infection.

Mice strains used for laboratory purposes for studying various infectious diseases are inbred population, which results in less heterogeneity and are generally genetically homogenous (Soldevilla et al., 2022). Several inbred strains of mice exhibit genetic variations that influence the outcome of the infection. The most commonly used mice strains are BALB/c, C57BL/6, C3HeB/FeJ, DBA/2 and 129/Sv. In general, mice are tolerant hosts and depending on which lineage is employed for the study, one can determine the degree of resistance to *Mtb* infections based on parameters like bacterial load, pathological damage and survival time (Mitsos et al., 2000; Eruslanov et al., 2005; Yan et al., 2006; Yang et al., 2021). BALB/c and C57BL/6 mice are more resistant to *Mtb* infection and demonstrate a mean survival time (MST) of more than 300 days to intravenous or aerosol *Mtb* infection (Medina and North, 1998; Beamer and Turner, 2005). On the other hand, C3HeB/FeJ, DBA/2 and 129/Sv mice are more susceptible to TB, displaying an MST of 150 days or less (Medina and North, 1998; Beamer and Turner, 2005). The differences in the susceptibility towards *Mtb* infection have been attributed to several genomic loci. Initial studies identified *Bcg (Ity, Lsh)* locus on mouse chromosome 1 to be involved in controlling the replication of antigenically unrelated intracellular pathogens in macrophages (Vidal et al., 1993; Malo et al., 1994). *NRAMP* (Natural resistance associated macrophage protein-1) was identified as one of the candidate genes belonging to the *BCG* locus. This gene encodes for a macrophage-specific transport protein, and a single mutation of glycine to aspartic acid at position 105 was observed to be associated with the susceptibility phenotype (Bcg^s^) in various inbred mouse strains (Vidal et al., 1993; Malo et al., 1994). Two distinct phenotypes exist: *BCG^s^* (susceptible) and *BCG^r^* (resistant) to infection with various pathogens including *Salmonella typhimurium*, *mycobacterial* species and *Leishmania donovani* (Plant and Glynn, 1976; Bradley, 1977; Gros et al., 1981; Goto et al., 1989). Later it was shown that NRAMP-1 does not play a role in protection against virulent *Mtb* challenge in various mice (Medina and North, 1996; Medina and North, 1998; North et al., 1999). Although the mutation in *NRAMP-1* does not influence *Mtb* infection, it has been shown that polymorphism in the human ortholog of *NRAMP-1* play a role in human TB susceptibility in certain populations (Bellamy et al., 1998; Cervino et al., 2000; Gao et al., 2000; Ryu et al., 2000).

By crossing the inbred strains and generating the F1 and F2 hybrids, many other genetic loci were mapped. Kramnik et al. identified a new locus, *sst1* (*super susceptibility to tuberculosis-1*), by analyzing the F2 hybrids (highly susceptible C3HeB/FeJ X C57BL/6 resistant mice), which mapped on chromosome 1 (Kramnik et al., 2000). This locus was found to be distinct from that of *Nramp1*. The *sst1* locus functions to control granuloma formation and necrosis in the lungs, reducing the pulmonary damage (Kramnik et al., 2000). Subsequent studies found *ipr1* gene to be present in *sst1* locus responsible for the resistance to *Mtb* infection (Pan et al., 2005). Moreover, Yan et al. demonstrated that *sst1* locus was associated with mechanisms that were independent of inducible NO synthase in innate immunity, rather than activation and migration of Th1 cells to the lungs (Yan et al., 2007). To extend the study in humans, polymorphisms in *SP110* (the human homolog of *lpr1* gene) were correlated with susceptibility to TB disease in various West African populations (Tosh et al., 2006; Chang et al., 2018). In another study, DBA/2 mouse strain, which is highly susceptible to *Mtb* infection, exhibits progressive bacterial replication, neutrophil-dependent pulmonary damage, extensive necrosis and finally early death. The susceptibility loci were identified to chromosome 1 (designated as Tuberculosis resistance locus (*Trl-1*), 3 (*Trl-2*) and 7 (*Trl-3*) by quantitative trait loci (QTL) mapping, that results in reduced MST on intravenous challenge (Mitsos et al., 2000). Subsequently, respiratory infection of F2 hybrids (DBA/2 x C57Bl/6) with *Mtb* H37Rv revealed a major locus on chromosome 19 (*Trl-4*) to be involved in bacterial replication in the lungs (Mitsos et al., 2003). These variations at the genetic level also lead to differences in pulmonary lesions and TB-associated pathologies among these various inbred mouse strains.

BALB/c and C57BL/6 (B6) mice have been infected through the natural route of infection i.e. via aerosols, by using inhalation chambers. High-dose of aerosol infection, usually implanting more than 5000 bacterial CFU per lung, causes death in these resistant mice within 3-4 weeks of *Mtb* challenge (Rhoades et al., 1997; Hunter et al., 2023). However, moderate or low-dose of aerosol infection i.e. implanting 500-1000 CFU or 50-100 CFU per lung, allows *Mtb* to replicate exponentially for the first 2-4 weeks post-infection. During this period, infection enters into the long chronic phase with controlled replication and limited pathological damage (Rhoades et al., 1997; Turner et al., 2001; Hunter et al., 2023). This phase usually lasts for around 6 months, after which mice eventually start to succumb to TB disease (Rhoades et al., 1997; Turner et al., 2001; Hunter et al., 2023).

One of the drawbacks of these two murine models is that they fail to develop necrotic and hypoxic granulomas like in human disease. Rhoades et al. demonstrated that B6 mice infected with *Mtb*, regardless of the dose of the inoculum showed similar pathological profile in the lungs (Rhoades et al., 1997). During the course of disease progression, the granulomatous response in all the mice, irrespective of the dose of infection, falls into five categories as described by Rhoades et al., with predominantly fused and foamy macrophages surrounded by loose aggregates of lymphocytes. As the infection progresses into the chronic phase, these loosely bound lymphocytes dissipate and spread across the entire lung section. The non-necrotizing small foci formed are characterized by interstitial fibrosis rich granulomas and thickened alveolar septae. Infact, even with high dose of infection, central necrosis was not observed, unlike in the case of granulomas observed in human patients with active TB (Rhoades et al., 1997).

Although BALB/c and B6 mice strains have been extensively employed for therapeutic testing of various anti-TB drugs and regimens, there are varying differences in the pathological responses, which do not coincide with the pulmonary lesions observed in human patient. This discrepancy leads to distinct effects of the various anti-TB drugs tested in these animal models. Microenvironments inside human granulomas, which are highly necrotic and hypoxic, influence the bacterial physiology and metabolism that can make the pathogen drug tolerant thereby impacting efficacy of the treatment (Driver et al., 2012; Irwin et al., 2016). Moreover, *Mtb* largely remains intracellular in mice granulomas in contrast to what is observed in human TB granulomas, where they are largely extracellular (Eum et al., 2010; Hoff et al., 2011). Thus, the efficacy of the drugs in terms of their ability to penetrate the fibrotic and necrotic centers, to work under hypoxic conditions and to show inhibitory effects on extracellular bacteria (which are metabolically distinct than intracellular bacteria) cannot be evaluated in these resistant models (Irwin et al., 2014).

Thus, the failure to develop necrotic and hypoxic granulomas has encouraged the use of other animal models for drug testing. The use of C3HeB/FeJ mouse model that was discovered by Igor Kramnik’s group is being encouraged for drug testing (Kramnik et al., 2000). These mice have genetic mutation in the interferon-inducible 75 (*ifi75*) gene, which is an isoform of *ipr1*, making them highly susceptible to *Mtb* infection (Pan et al., 2005). C3HeB/FeJ mice develop large, caseating granulomas in the lungs, which are hypoxic following low dose aerosol infection with *Mtb* (Kramnik et al., 2000; Harper et al., 2012). Moreover, these lesions contain abundant extracellular bacilli and are enclosed by foamy macrophages containing intracellular bacilli (Driver et al., 2012; Harper et al., 2012). Infact, as the disease progresses, these lesions also show fibrosis (Harper et al., 2012). Thus, all these pathological features resemble human pulmonary lesions and represent conditions that prevail in human TB. Moreover, efficacy of various clinical anti-TB drugs were compared in both BALB/c and Kramnik mice infected with *Mtb* (Driver et al., 2012). It was observed that Kramnik mice exhibited less efficacy to monotherapy with drugs such as isoniazid (INH), rifampicin (RIF), linezolid (LZD), or pyrazinamide (PZA) than BALB/c mice. More than 99% bacteria were eliminated from BALB/c mice following PZA treatment, while no reduction in bacilli number was observed in C3He/FeJ mice (Driver et al., 2012). Further, *Mtb* infection of NOS2-/- deficient mice that are incapable of producing nitric oxide (NO) in immune cells also developed necrotic and hypoxic granulomas (Gengenbacher et al., 2017). While INH treatment of these infected mice induced drug-tolerant population with the start of the necrotic lesions, other drugs, such as pretomanid, delamanid and BTZ043 showed bactericidal activity independent of pulmonary pathology (Gengenbacher et al., 2017). Thus, these susceptible models represent a realistic tool for assessing various anti-TB drugs due to their ability to represent TB lesions pathologically similar to humans.

### 2.2 Guinea pigs

Guinea pigs (*Cavea porcellus*) represent one of the closest models to humans to study TB related pathology. During the 19^th^ century, these small animals were employed to study TB and diphtheria (Padilla-Carlin et al., 2008). Infact, the famous Koch postulates were developed by using guinea pig model and are now considered essential prerequisites for identification of the causative agent of any infectious disease (Padilla-Carlin et al., 2008). Apart from using guinea pigs as model for TB, they have been widely used to study other infectious diseases such as sexually transmitted diseases, infections related to *Staphylococcus aureus* and *Legionnaires* disease (Padilla-Carlin et al., 2008). Moreover, the causative agents of both TB and diphtheria were identified by using guinea pigs, efforts of which led to Noble Prizes (Koch, 1882; Behring, 1890).

Guinea pigs share a number of immunological and hormonal features with humans, including pulmonary structure and physiology, response to corticosteroids, requirement for an exogenous supply of vitamin C and delayed-type hypersensitivity (DTH) reaction after getting exposed to any infection (Gell and Benacerraf, 1961; Claman, 1972; Ganguly et al., 1976; McMurray, 1994; Meurs et al., 2006; Padilla-Carlin et al., 2008). While gene technology advancements can produce gene knockout or knock-in lines easily in murine model, such protocols are not available for guinea pigs. Besides, the myriad of immunological reagents that are readily available for mice are unavailable for guinea pigs. Nevertheless, other methodologies including various bioassays, antibody blocking and molecular techniques to study relative gene expression are being developed to learn about various components of immune response (Kawahara et al., 2002; Yamamoto et al., 2002; Jeevan et al., 2003; Yamada et al., 2005; Banasik et al., 2019). Infact, the importance of CD1-restricted T cells in *Mtb* infections were identified in guinea pigs (Hiromatsu et al., 2002). Jain and Dey et al. developed an oligonucleotide microarray in guinea pigs to study the global transcription profile to identify disease specific signatures for pulmonary TB (Jain et al., 2012). Ordway and the group used quantitative polymerase chain reaction (qPCR) along with flow cytometry to monitor changes in lymphocyte populations in guinea pigs (Ordway et al., 2007). The group also showed that clinical strains of *Mtb* elicit different kind of immune responses, especially the induction of regulatory T cells by highly virulent strains in comparison to laboratory strains (Shang et al., 2011). Despite these tools, the immunological reagents required to monitor immune responses to vaccine candidates in guinea pigs are limited, confining their use towards protection studies (Clark et al., 2015). Nevertheless, guinea pigs remain a popular model for studying TB primarily because they are highly susceptible to TB and can get infected with aerosols at very low dose (10-30 bacilli), mimicking the human situation. During the first 3-4 weeks of infection, *Mtb* progressively grows in guinea pig lungs (active phase) before the onset of adaptive immunity and subsequent containment of the bacterial replication (Smith et al., 1970; Turner et al., 2003). Granulomas are a trademark of TB disease that defines the outcome of the infection. Early during the infection, guinea pigs show small lesions in their lungs, often situated near the airways that consist of mainly macrophages, neutrophils and few lymphocytes (Hunter et al., 2023). At this stage, typically two weeks post-infection, lymphangitis is also observed in infected guinea pigs (Basaraba et al., 2006). By 15-20 days of infection, a typical granuloma in guinea pigs is developed consisting of epithelioid and foamy macrophages (Turner et al., 2003). This is further encapsulated by layers of lymphocytes mainly CD4+ and CD8+ T cells and subsequently calcification takes place to contain the infection and prevent bacterial spread (Turner et al., 2003). Unlike the mouse model, where the distribution of CD4+ and CD8+ T cells is discrete, these T cells are situated at the periphery in guinea pig granulomas. Finally, by 4 weeks of post-challenge, central necrosis is evident with many extracellular bacteria (Ordway et al., 2007; Hunter et al., 2023) As the disease progresses with time, infiltration of various inflammatory cells in the granulomas lead to replacement of normal lung parenchyma disrupting the local blood supply and compression of adjacent blood vessels, which lead to tissue hypoxia (Via et al., 2008; Orme and Basaraba, 2014). With time, the central necrotic core begins to show signs of wound repair, including fibrosis and dystrophic calcification (Orme and Basaraba, 2014). These remarkably similar pathological features shared with human TB disease, make guinea pigs one of the most suitable models for drug testing and vaccine efficacy assessment.

Guinea pigs are considered the gold standard for TB vaccine evaluation and serve as an initial guide. Infact, almost every vaccine candidate has been tested for its protective efficacy in this animal model before entering any clinical set up. The efficacy of various vaccine candidates and regimens can be easily assessed in guinea pigs by monitoring the reduction in the bacterial counts in the lung and spleen as well as changes in pathological damage (Williams et al., 2009). Moreover, weight loss is an indicator of the disease progression and survival time is one of the most significant parameter for vaccine efficacy (Williams et al., 2009). According to the EU cluster of TB vaccine evaluation forum, three doses of aerosol challenge are employed for vaccine efficacy testing (Williams et al., 2005). Under low dose aerosol challenge i.e., 5-10 bacilli per lung, the MST of guinea pigs infected with virulent *Mtb* is around 20 weeks (Reddy et al., 2012; Khare et al., 2013), whereas medium dose of infection i.e., ~20-50 bacilli per lung leads to rapid progression of the disease and animals succumb to TB by 30 weeks (Brandt et al., 2004; Williams et al., 2009). While high dose challenge of ~ 500-1000 bacilli, animals die within 8 to 20 weeks (Williams et al., 2005; Clark et al., 2015). Although high dose is not clinically relevant, there are few vaccines, which have been tested at this dose (Jain et al., 2008).

The main focus of evaluating the protective efficacy is to show that the new vaccine candidate imparts better protection than BCG control in guinea pigs. Many different kinds of vaccines are developed based on various approaches, such as recombinant BCG, live attenuated vaccines, DNA vaccines, subunit vaccines and atypical mycobacterial strains. Several of these vaccine candidates, which are being assessed in various stages of clinical development were initially tested for their protective efficacy in guinea pigs (Brandt et al., 2004; Martin et al., 2006). In general, BCG is employed as positive control in every efficacy experiment, which reduces the bacterial counts significantly by 1.5 to 2.0 log_10_ and effectively prolongs the survival time in comparison to unvaccinated control under low-dose challenge (Grover et al., 2009; Nangpal et al., 2017). Thus, any new vaccine candidate that shows better efficacy in comparison to BCG vaccination stands a chance to go further for downstream development.

In addition, various *Mtb* knockout strains are employed for infection of guinea pigs, not only to study their role in virulence and survival of the pathogen, but also to validate these virulence factors as important drug targets. Many *Mtb* genes involved in cell wall biosynthesis, DNA related processes, acidic resistance genes, energy metabolism, evasion of host immunity and iron uptake systems etc., have been validated *in vivo* in guinea pigs as crucial drug targets (Singh et al., 2003; Reddy et al., 2013; Chai et al., 2020). Guinea pigs also played an important role in identifying several auxotrophic *Mtb* mutants that were shown to be attenuated in these animals, as potential vaccine candidates. For instance, *bioA* mutant of *Mtb*, administered either via aerosols or intradermally, shows severe attenuation in guinea pigs (Kar et al., 2017). Immunization of guinea pigs with *Mtb*ΔbioA induced significant protection against an *Mtb* challenge, when compared with the unvaccinated animals (Kar et al., 2017). Likewise, *Mtb*ΔleuDΔpanCD auxotrophic strain, carrying two independent deletions in the essential *leuD* (Rv2987c) and *panCD* (Rv3602c and Rv3601c) loci, was shown to be immunogenic and protected guinea pigs against aerosol challenge of *Mtb* (Sampson et al., 2004).

Thus, to summarize, guinea pigs have provided us with valuable information about the pathogenesis of *Mtb* as well as virulence determinants of the pathogen. They have served as an initial platform for evaluation the efficacy of many new vaccines candidates and development of new immunological reagents and tools will help in increasing the usage of guinea pigs in future research.

### 2.3 Non-human primates

Non-Human Primates (NHPs) hold an evolutionary significance and share similar characteristics of TB disease to those observed in humans, making them the most reliable choice to evaluate TB vaccines. However, most of the new vaccines that are tested in NHP model are initially screened in guinea pigs and mouse for preliminary evaluation to eliminate the non-promising candidates that do not show their worth in small animals. Very few laboratories worldwide employ NHP model for conducting TB-related studies, primarily because of the high maintenance costs, handling issues, ethical limitations and the mandatory need for biocontainment facility. Different NHP species exist that are employed for studying various aspects of TB research. The clinical spectrum of TB infections in humans is vast and can be observed in NHP model as different animals show varied outcomes, including latent infection, dissemination and pneumonia (Laddy et al., 2018). Infact, the granulomas observed in NHPs also become caseous with further liquefaction and subsequent cavitation (Laddy et al., 2018). Rhesus macaques (*Macaca mulatta*, RM) and Cynomolgus macaques (*Macaca fascicularis*, CM) are the two most commonly used NHP species for TB research. Most of the initial TB studies in the Golden Age in 1970s, were conducted in Indian RMs, which were focused on understanding the efficacy and immunogenicity of BCG vaccine (Barclay et al., 1970; Baram et al., 1971). Infact, there was one study which showed the use of ethambutol and isoniazid as TB drugs in RMs (Schmidt, 1966). After two decades of dedicated research on RMs, the use of CM was identified for TB-related studies, and since then, extensive research has been conducted on these two species to assess efficacy of novel TB vaccines, determine the therapeutic efficacy of new anti-TB drugs and understand TB pathogenesis in great detail.

These old-world monkeys (RM and CM) have been known to get infected with *Mtb* and develop human-like disease, however, the new world monkeys (*Callithrix jacchus*, the common marmoset) are also now employed for studying TB pathology (Via et al., 2013). These monkey strains vary in their susceptibility to *Mtb* infections and clinical outcomes. RM are more susceptible to *Mtb* infections than CM, which develop both active as well as latent tuberculosis (Maiello et al., 2018). Moreover, ultra-low-dose aerosol *Mtb* challenge along with stereological techniques to determine bacterial burden also demonstrated that RMs are more susceptible than CMs (Sharpe et al., 2016). The very first report of using CM was published by Walsh et al. in 1996, which showed that high dose of intratracheal *Mtb* challenge led to progressive fatal infection in Philippine cynomolgus monkey (Walsh et al., 1996). On the other hand, low-dose of challenge developed a chronic, slowly progressive, localized form of pulmonary TB with significant number of CMs able to contain the infection (Walsh et al., 1996). Later, low-dose challenge (10-25 CFU of *Mtb* Erdman) administered intrabronchially resulted in active disease in ~50% of CM animals, while remaining half of the animals showed no evidence of disease for 15-20 months of the study and remained clinically latent (Capuano et al., 2003; Lin et al., 2009). Infact, the same group, in their subsequent studies demonstrated that LTBI monkeys could reactivate on treatment with anti-tumor necrosis factor (anti-TNF) or depletion of CD4+ T cell or simian immunodeficiency virus (SIV) coinfection (Diedrich et al., 2010; Lin et al., 2010; Lin et al., 2012b).

Although RMs are highly susceptible to *Mtb* infections, one of the reports show evidence of latent infection in these species (Gormus et al., 2004). It was observed that RMs infected either via the aerosols or through intrabronchial instillation show similar clinical outcomes in terms of disease burden, however, exhibit variations in the pathology (Sibley et al., 2016). Both RM and CM have been employed for TB vaccine studies. It was shown that BCG vaccination of CM provided better protection to animals against *Mtb* infection in comparison to BCG immunized RM (Langermans et al., 2001). Infact, the worth of MVA85A vaccine as a booster vaccine and SO2 vaccine strain (live attenuated, phoP deficient *Mtb* mutant strain) was observed in RMs. The study showed that both the regimens involving either the SO2 strain or BCG-primed RMs boosted with MVA85A showed significant protection with reduced pathological damage and chest X-ray scores against *Mtb* Erdman challenge (Verreck et al., 2009). Moreover, the most advanced TB vaccine *MTB*VAC, which is also live attenuated *Mtb* based vaccine, was tested in RMs. A single intradermal administration of *MTB*VAC resulted in protection of these animals against aerosol challenge of *Mtb* as observed by significantly reduced pathological damage as shown by *in vivo* medical imaging, gross pathology lesion counts and pathology scores (White et al., 2021). Moreover, the immune parameters measured matched the profile that were determined in humans vaccinated with *MTB*VAC (White et al., 2021).

CMs have also been employed for TB vaccine studies especially in context to reactivation TB. For instance, multistage vaccine H56, is a subunit-based vaccine comprising of Ag85B and ESAT-6 (acute phase secretory *Mtb* proteins) fused with Rv2660c (the nutrient stress-induced antigen), given along with adjuvant IC31, was able to boost the protection of BCG-primed CMs by significantly reducing the clinical disease progression against *Mtb* challenge in CM. It was also observed that reactivation of latent infection was prevented in these vaccinated CM (Lin et al., 2012a). Another subunit vaccine M72f, formulated with AS02A adjuvant was evaluated in CMs (Reed et al., 2009). Animals immunized in BCG priming and boosting with M72f/AS02A vaccine regimen imparted better protection in comparison to BCG alone vaccination against *Mtb* challenge as measured by pathological assessment and survival time (Reed et al., 2009).

Apart from being employed for TB vaccine efficacy and immunogenicity studies, macaques are also being used for studying various aspects of TB pathogenesis, including factors that contribute to reactivation TB and various important host-pathogen interactions. For instance, transcriptomics of TB granulomatous lesions of RM with active TB was performed and it was found that early stages of infection are characterized by high levels of immune pathways related to proinflammatory cytokines, which are indeed required to contain immunopathological damage. However, during the late stages, lesions showed reduced inflammatory responses (Mehra et al., 2010). Such host profiles can be helpful in identifying markers of latency and reactivation (Mehra et al., 2010). Moreover, macaques have been successfully employed to identify important genes of *Mtb* that play crucial role in the virulence of the pathogen. *Mtb* SigH is a stress-induced transcriptional factor that is important for the pathogens survival under various *in vitro* conditions including heat, oxidative-stress, envelope damage and hypoxia. *Mtb*ΔSigH mutant strain was found to generate stronger innate responses in bone marrow derived macrophages (BMDMs) isolated from RMs in comparison to wild type *Mtb* (Dutta et al., 2012). In their subsequent study, NHPs were challenged with *Mtb* or *Mtb*ΔSigH mutant and the disease progression was monitored in both groups by using clinical, pathological, microbiological, and immunological parameters (Mehra et al., 2012). It was observed that NHPs infected with *Mtb* alone exhibited higher bacillary load along with granulomatous immunopathological damage. Moreover, all the animals rapidly succumbed to TB disease. On the contrary, NHPs exposed to the *Mtb*ΔSigH mutant did not exhibit acute tuberculosis and survived the entire duration of the study (Mehra et al., 2012), thus, validating the importance of SigH in providing survival advantage to pathogen *in vivo*, making it a potential drug target.

Macaques are also an excellent model to study TB/SIV (simian immunodeficiency virus) coinfection, especially in context to understanding reactivation TB, since people with HIV and latent TB have higher risk of developing TB in comparison to HIV-negative latently infected individuals (Mukadi et al., 1993; Post et al., 1995). SIV infection serves as a counterpart to HIV in NHPs and have been used in number of studies employing both RM and CM. For example, SIV infection of all latently infected CM induced reactivation TB, with a variable time to reactivation (up to 11 months post-SIV), which was correlated to depletion of peripheral T cells during acute SIV infection rather than viral load (Diedrich et al., 2010). Similarly, *Mtb* infection of RM (~500 CFU *Mtb* CDC1551 via a head-only aerosol method) induced latent aymptomatic infections in these NHPs. Coinfection of the latently infected RMs with SIV significantly induced reactivation TB and showed significantly higher body temperature, CRP levels and body weight loss than the *Mtb* monoinfected group (Mehra et al., 2011). In another study published by Mattila et al., the group investigated multifunctional T cell profile and granuloma T cell responses in reactivated TB in CM model of HIV–*Mtb* coinfection (Mattila et al., 2011). They found differences in the multifunctional T cell responses in animals showing reactivation <17 weeks than in animals that reactivated >26 weeks (Mattila et al., 2011).

Thus, to recapitulate, macaques offer several advantages for being the closest to humans anatomically and physiologically. Besides, the remarkable similarities they share with human TB, representing the wide spectrum of clinical manifestations, makes these animals the most suitable for conducting TB vaccine efficacy as well as TB drug screening studies. HIV/TB coinfection is also a major health concern, and while small animals can be infected with *Mtb*, they are not the hosts for HIV infections and hence, NHPs represent an excellent model for studying TB and AIDS coinfections.

### 2.4 Rabbits

Rabbits (*Oryctolagus cuniculus*) have been used historically for studying various human diseases, including syphilis, TB, HIV-AIDS and acute hepatic failure etc. In 1920, Lurie and his team worked extensively on these animals to study the immunopathogenesis of TB by inbreeding various susceptible and resistant strains of rabbit and defined various pathological features, which were human like from formation of granulomas to caseous necrosis and cavitation (Lurie, 1928; Dannenberg, 2001; Dannenberg, 2009). However, these lines of rabbits no longer exist and have become extinct. The most common rabbit breed available now is the New Zealand White (NZW) rabbits, which have also been employed as models to study various aspects of TB pathology, latent tuberculosis, spinal TB and TB meningitis (Flynn et al., 2008; Manabe et al., 2008; Kaplan and Tsenova, 2010; Yue et al., 2022; Esteves et al., 2018). In general, the outbred strains of rabbits are more resistant to *Mtb* and recover within 4 to 6 months of infection (Heppleston, 1949; Lurie et al., 1952). Infact, rabbits are more resistant to *Mtb* infection in comparison to guinea pigs and mice (Gupta and Katoch, 2005). While these outbred New Zealand white rabbits are naturally resistant to *Mtb* infections, *M. bovis* infection leads to cavitary disease, which is fatal (Converse et al., 1996). The response of NZW rabbits to infection varies with *Mtb* strains being used (Manabe et al., 2003). One of the studies compared the three different strains of *Mtb*: Erdman, H37Rv, and CDC1551 for their ability to cause disease in rabbits (Manabe et al., 2003). It was observed that *Mtb* Erdman was the most virulent strain, which required the least number of bacteria to form a visible tubercle at 5 weeks post-infection in comparison to H37Rv. While most of the rabbits infected with *Mtb* H37Rv recovered within 4 to 6 months, several Erdman infected rabbits showed caseous tubercles with two of the rabbits having cavitary lesions (Manabe et al., 2003). Rabbits infected with HN878, a hyper-virulent *Mtb* strain, show heterogeneous lesions within the same animals as observed in humans (Flynn et al., 2008; Subbian et al., 2011b). CDC1551, a clinical isolate of *Mtb*, causes latent infections in rabbits, although these animals did not show any evidence of spontaneous reactivation unless immunosuppressants like corticosteroids were administered (Subbian et al., 2012). In addition, inbred strains of NZW rabbits are more susceptible to infections in comparison to outbred rabbits, giving an opportunity to understand the mechanisms underlying resistance to TB (Dorman et al., 2004). Unlike the murine model, necrotic granulomas observed in rabbit lungs also show evidence of hypoxic microenvironment (Via et al., 2008). Thus, rabbits represent a suitable model for TB related studies as it can mimic various prominent pathological features of human TB.

Rabbits infected with HN878 strain leads to TB disease with similar pathological characteristics as humans and thus, are used for studying kinetics, penetration and distribution of standard anti-TB drugs (Kjellsson et al., 2012; Via et al., 2012; Rifat et al., 2018). In one of the studies, by using onlinear mixed-effects pharmacokinetic modeling approach, it was found that INH, RIF and PYZ were much less concentrated in lesions than in plasma, while moxifloxacin was able to very well partition into lung and granulomas (Kjellsson et al., 2012). A study published by Rifat et al. measured the tissue drug distribution and penetration ability of two rifamycin antibiotics (rifampin and rifapentine) by using pharmacokinetic-pharmacodynamic (PK/PD) modeling (Rifat et al., 2018). They used rabbit model of experimental TB to show that both drugs were able to penetrate different lung lesions as well as the fibrotic wall of cavitary lesions and exhibited a penetration coefficient of ≥1 when compared to plasma (Rifat et al., 2018). However, rifampin was able to penetrate the necrotic core i.e., caseum much efficiently than rifapentine (Rifat et al., 2018). These studies also correlated well with clinical data wherein patients with cavitary lung lesions were not benefitted from rifapentine treatment (Rifat et al., 2018). Rabbits have also been employed to study the effect of immune modulation on treatment outcomes (Subbian et al., 2011a; Datta et al., 2015).

While rabbits are a model systems for studying pulmonary TB, they are also explored to understand pathogenesis of extrapulmonary TB, including spinal TB and TB meningitis (Tsenova et al., 1998; Jia et al., 2024). Although the rabbit model is considered suitable for conducting TB research, the limited availability of validated immune reagents and the requirement of larger biocontainment facility restricts its use. **Table 1** summarizes the features of various animal model systems employed for TB research, highlighting their advantages, disadvantages and their applications.

**Table 1:** Animal models employed for TB research: *pros and cons*.

Animal Model	Strains available	Advantages	Disadvantages	Applications	References
Mouse (Mus musculus)	Resistant strains: • BALB/C • C57BL/6 Susceptible strains: • C3HeB/FeJ, • DBA/2 • 129/Sv	• Cost effective • Ease of handling • Availability of knock out mice strains • Availability of immunonological reagents • Limited capacity required for housing	• Differences in the TB associated pathological features such as granuloma structure which is different in mouse as compared to human. • Do not form necrotic cores in TB lesions unlike human TB granulomas except in C3HeB/FeJ model	• TB immune response studies • Drug efficacy studies • PK-PD studies • Vaccine efficacy and immunogenicity studies • Vaccine safety studies	(Rhoades et al., 1997; Medina and North, 1998; Kramnik et al., 2000; Beamer and Turner, 2005; Pan et al., 2005; Eum et al., 2010; Hoff et al., 2011; Driver et al., 2012; Harper et al., 2012)
Guinea Pigs (Cavea porcellus)	• Dunkin Hartley	• Cost effective • Highly susceptible to tuberculosis infection • Form necrotic cores in granulomas similar to that in humans	• Non availability of immunological reagents thus, are not extensively used for studying immune parameters • Does not develop latency	• Vaccine efficacy studies • Studies related to identification of *Mtb* virulence determinants	(Turner et al., 2003; Via et al., 2008; Orme and Basaraba, 2014; Hunter et al., 2023)
Non-Human Primates	• Rhesus macaques (Macaca mulatta) • Cynomolgus macaques (Macaca fascicularis) • Callithrix jacchus, the common marmoset	• Shows similar clinical outcomes to TB infections as humans • Share similar immune response and pathological features like that observed in human TB • Can develop LTBI on *Mtb* infections	• High maintenance costs • Handling issues • Ethical limitations • Requirement of large housing capacity	• Evaluation of efficacy of novel TB vaccines • Evaluation of therapeutic efficacy of new anti-TB drugs • Studies related to TB pathogenesis • TB-HIV co-infection studies	(Barclay et al., 1970; Baram et al., 1971; Gormus et al., 2004; Diedrich et al., 2010; Mattila et al., 2011; Mehra et al., 2011; Via et al., 2013; Sibley et al., 2016; Laddy et al., 2018; Bucsan et al., 2019)
Rabbits (Oryctolagus cuniculus)	• New Zealand White (NZW)	• TB granulomas form necrotic cores and are hypoxic • Evidence of liquefaction and cavitation as observed in human TB	• High maintenance costs • Resistant to laboratory strains like H37Rv and Erdman • Requirement of large housing and bio- containment facility	• Drug and vaccine efficacy studies • Studies related to TB pathology and latent tuberculosis, • Model to study spinal TB and TB meningitis	(Tsenova et al., 1998; Manabe et al., 2003; Via et al., 2008; Subbian et al., 2011b; Jia et al., 2024)

## 3 Omics-based approaches to understand pathogenesis via animal models

**Figure 2:**
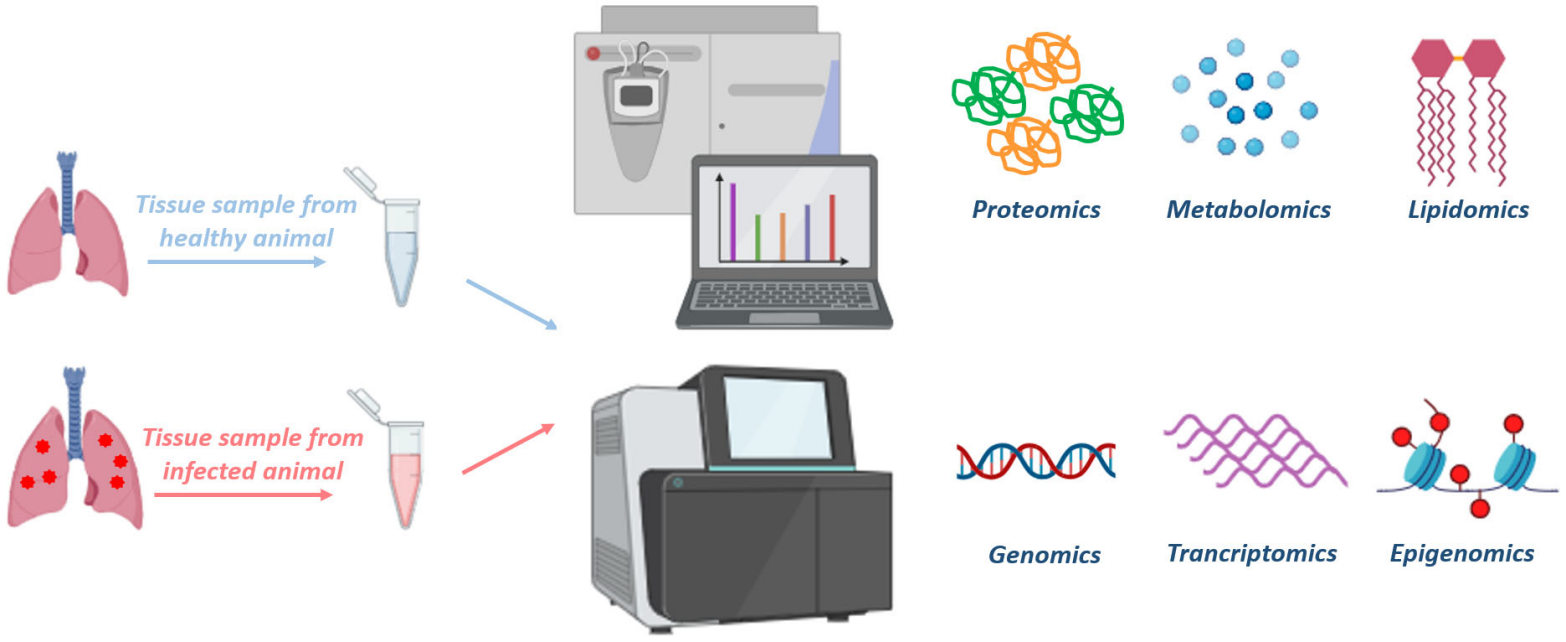
Multiple omics-based approaches to understand disease biology: Various tissue samples can be collected to isolate different biological molecules (DNA, RNA, metabolites, proteins and lipids) from animals. These tissue samples can be processed and subjected to mass spectrometry or NGS platforms to perform various omics-based approaches, such as proteomics, metabolomics, lipidomics, genomics, transcriptomics, epigenomics, respectively. Analysis of the omics data allow us to compare the differences between the disease progression and characteristics of healthy vs diseased or treated vs untreated tissue samples. Moreover, integration of multi-omics data would help us in understanding the complete spectrum of the disease, allowing the discovery of therapeutic targets and disease-biomarkers. The figure is prepared by using BioRender.com.

Omics-based technologies are approaches, which aim to measure and evaluate an ensemble of biomolecules together in order to understand the contribution of each molecule under investigation. They offer the advantage of being unsupervised, making them a less biased methodology that can reveal new insights into the query being addressed. Genomics, transcriptomics, proteomics, metabolomics and lipidomics are some of the commonly employed techniques which allow researchers to delve into the fundamentals of infection biology and pathogenesis (**Figure 2**). Besides, these techniques can be used to explore molecular mechanisms underlying a disease, understand the basis of drug resistance and also host response to an infection (Khan et al., 2019).

In addition, omics-based technology provides avenues for identifying biomarkers with therapeutic or diagnostic potentials, for rational drug designing and for developing novel vaccines (Ahamad et al., 2022; Guha et al., 2024). In particular, discovery of host signatures by comparing diseased versus uninfected groups has helped to identify diagnostic biomarkers (Burel et al., 2019). Such study designs have provided opportunities to explore host genes, proteins, metabolites or pathways that get altered upon infection, thereby gaining mechanistic understanding of the disease and providing a platform for designing host-directed therapeutics. Besides, these approaches have also been employed to elucidate global alterations that take place in the host post-treatment with a drug, giving a holistic view with regard to the mechanism of action of the molecule and revealing new insights into drug activity.

*Mtb* infections are complex, with the host undergoing various changes at genomic, transcriptomic, and proteomic levels during the course of the disease. Additionally, the host deploys various metabolic strategies to limit the supply of nutrients to the pathogen and, in turn, limit bacterial growth. Although, to understand TB pathogenesis, *in vitro* models have been developed to represent various features of the *in vivo* conditions, such as low oxygen levels (Rustad et al., 2008), low nutrients (Betts et al., 2002) and the addition of exogenous stresses (Stewart et al., 2002; Deb et al., 2009). However, they cannot fully mimic the microenvironment that *Mtb* faces inside host cells. While macrophages have been employed for several *ex-vivo Mtb* infection experiments, the absence of interacting immune cells (eg. T cells, natural killer cells, dendritic cells) as well as the lack of the ability to form a granulomatous structure are major shortcomings of using this cell culture system (Schnappinger et al., 2003; Cappelli et al., 2006; Rachman et al., 2006a; Fontán et al., 2008; Tailleux et al., 2008; Ward et al., 2010). It is convincing to believe that *in vivo* models can closely mimic the multifarious milieu as seen in human lungs. Thus, conducting large-scale expression profiling experiments using animal models is a rational approach to identify genes required for pathogen clearance.

Transcriptomics allows quantifying the abundance and differential expression of various transcripts of an organism exposed to different conditions. Understanding the transcriptome is the key for connecting information attained from genomics to protein target expression (Talaat et al., 2004; Rachman et al., 2006b; Talaat et al., 2007). This can be applied for the identification of responses to infection or to drug exposure, and further, characterize potentially druggable pathways. Additionally, RNA profiling of both the pathogen and the host lays the basis for understanding interactions at the host-pathogen interface (Westermann et al., 2017). Host transcriptomics is one of the leading approaches to discover immune signatures between uninfected and infected host samples, which can pave the way for identification of novel biomarkers. In addition, transcriptomics also allows detection of infection-associated antigens, such as circulating and secreted host RNA (miRNA, lncRNA), blood cell-produced RNA or bacterial secreted RNA (van den Esker and Koets, 2019). Transcriptome analysis of blood samples isolated from C3HeB/FeJ mice infected with HN878 strain of *Mtb* revealed a signature that was associated with high type I interferons, activation and recruitment of neutrophils and a reduction in B lymphocyte, NK cells and T-cell effector responses, all of which leads to TB in humans (Moreira-Teixeira et al., 2020).

In another study, RNA sequencing was employed to determine the changes that occur in the host transcriptome upon *Mtb* infection. The authors had demonstrated that *Mtb* strain lacking the MenT3 and MenT4 toxins (*MtbΔment3Δment4)*, displayed severe attenuation in BALB/c mice as well as in guinea pigs (Gosain et al., 2024). Detailed host RNA-seq analysis of lung tissues, revealed increased transcripts levels of proteins that were associated with calcium signaling, apoptotic pathway and autophagy in mice infected with the mutant strain, as compared to wild type infected mice. Moreover, inflammatory responses were much reduced in animals infected with the *MtbΔment3Δment4* mutant (Gosain et al., 2024). Thus, *in vivo* RNA sequencing identified differentially expressed genes upon infection with *Mtb* and help in elucidation of the possible mechanisms behind the attenuation of the mutant strain inside the host (Gosain et al., 2024).

Since *Mtb* infection leads to alterations in the host metabolome, characterization of such changes is important as it may result in identification of host-protective pathways and thus, aid in the development of host-directed therapies (Vrieling et al., 2020). Metabolomics offers advantages for the identification of low molecular weight metabolites (catabolites as well as anabolites) that are altered in response to various pathophysiological events in both *in vitro* and *in vivo* animal models, as well as in human patients (Frediani et al., 2014; Lau et al., 2015; Borah et al., 2021). The identified metabolites can be employed as biomarkers for diagnosis or trustworthy markers that can distinguish between unhealthy or healthly status, and for the evaluation of potential therapeutics.

In another study, TB granulomatous lung tissues were employed to evaluate the metabolic status with respect to host response in guinea pigs infected with low-dose *Mtb* H37Rv infection. Metabolite profiling performed by using 1H HRMAS (high resolution magic angle spinning) NMR spectroscopy led to the unambiguous identification of 20 distinct host metabolites involved in various cellular pathways, such as anaerobic glycolysis and TCA cycle as the infection progressed (Somashekar et al., 2011). Lactate is produced by anerobic glycolysis and its levels were found to increase from 15 days onwards (Somashekar et al., 2011). Accumulation of lactate can be considered as an index of hypoxia developed inside the granulomatous tissue as well as host tissue necrosis (Via et al., 2008; Kraut and Madias, 2014). Similarly, the increasing levels of reduced glutathione (GSH) were also observed which is considered as a measure of oxidative stress as the disease progresses. Moreover, the GSH redox system is one of the most important antioxidant defense systems for maintaining redox homeostasis in lung cells (Venketaraman et al., 2005). This study also suggested that *Mtb* utilizes host lipids for *in vivo* growth as evident from the increased levels of phosphocholine (PC), glycerophosphocholine (GPC) and a concomitant depletion of phosphatidylcholine (PtC) observed with the disease progression, indicating that free fatty acids so produced could act as carbon and energy source for intracellular *Mtb* metabolism (Somashekar et al., 2011).

Further, global metabolic changes were studied in *Mtb* H37Rv infected C57BL/6 mice by extracting metabolites from the infected lungs at 4 weeks and 8 weeks post-infection (Fernández-García et al., 2020). This untargeted MS-based lung metabolomic study revealed that high levels of trimethylamine-N-oxide (TMAO) may be undesirable for the host and may probably impact *Mtb* growth positively. Similarly, increased levels of kynurenine was also observed, which is a tryptophan degradation metabolite involved in immunomodulation. This may again exert deleterious effects on the host immune system, as it is known that inhibiting the activity of indoleamine 2,3-dioxygenase (IDO) promotes TB infection control (Routy et al., 2016; Gautam et al., 2018). A shift in metabolism towards fatty acid oxidation was observed, as evident from the depleting carbohydrates at 4-week time point as well as increasing carnitines at 9-week time point. This shift describes the ability of *Mtb* to modify host macrophages from an inflammatory phenotype to an anti-inflammatory one (Huang et al., 2015; Gaber et al., 2017). Apart from these, metabolites pertaining to amino acids, polyamines and oxido-reductive stress were also found to be modulated upon *Mtb* infection in the host (Fernández-García et al., 2020).

In another study, 1H NMR-based metabolomics was employed to conduct global profiling to characterize the responses induced in C57BL/6 mice upon virulent *Mtb* infection (Shin et al., 2011). Various metabolites associated with amino acid and nucleotide metabolism, membrane phospholipids, glycolysis and the antioxidative stress elements were found to be altered in the host upon infection. Glycogen and glucose levels were observed to be decreased, while lactate levels were elevated in the infected mice. *Mtb* can survive in a low glucose environment because it can interplay the carbon source between the β-oxidation of lipids and the glyoxylate pathway to replenish the TCA cycle intermediates (Sharma et al., 2000; Lorenz and Fink, 2001; Serafini et al., 2019). Indeed, succinate was found to be increased significantly in the organs of *Mtb* infected mice when compared to the naïve mice. Intermediates of pyrimidine and purine metabolism were found to be increased in infected mice, suggesting that active cell division takes place in *Mtb* infected organs, especially in the lungs (Reyes et al., 1982; Simmonds et al., 1997; Berg et al., 2002; Chaudhary et al., 2004; Daniel et al., 2007; Hyde, 2007; Weljie et al., 2007). Further, this was the first report to demonstrate that itaconate was increased in the lungs infected with *Mtb*. Though the reason remains unclear, it was suggested by the authors that it can inhibit isocitrate lyase, which acts as a main enzyme in the glyoxylate pathway of *Mtb* (Hillier and Charnetzky, 1981; Berg et al., 2002; Daniel et al., 2007; Shin et al., 2011).

A NMR-based metabolite profiling of lung tissues of guinea pigs infected with *Mtb* W-Beijing strains led to the identification of 16 metabolites involved in carbohydrate, membrane lipids and amino acid metabolisms that were altered (Somashekar et al., 2012). For instance, levels of lactate, choline compounds, nicotinamide, and glutamate were significantly reduced, while formate and acetate were shown to be high in the infected serum samples (Somashekar et al., 2012). The observed signatures were attributed to hypoxic TB lesions, the Warburg effect or the production of free radicals in response to infection (Venketaraman et al., 2005; Lenaerts et al., 2007; Dakubo and Dakubo, 2010; Somashekar et al., 2012; Qualls and Murray, 2016).

It has been reported that treatment with methionine sulfoximine (MSO), an irreversible inhibitor of glutamine synthetase enzymes, leads to a reduction in the bacterial load in the *Mtb* infected guinea pigs (Harth and Horwitz, 2003; Tullius et al., 2003; Harth et al., 2005). Evidences of JHU083, a glutamine (Gln) metabolism antagonist drug, has been shown to reprogramme the host immune-metabolic signatures as well as improve effector T-cell responses in various murine tumor models (Rais et al., 2016; Leone et al., 2019; Oh et al., 2020). These observations, led to the hypothesis that JHU083, apart from directly possessing antibacterial activity, may also serve as a host-directed therapy against TB by reprogramming Gln metabolism. Further, it was shown that administration of JHU083 reduced the lung bacillary load by 1.9 log_10_ CFU at 5-weeks post-treatment compared to untreated mice, and consequently prolonged the survival of animals significantly. Moreover, LC/MS-based metabolomics of total lung tissues from untreated, JHU083-treated and RIF-treated animals led to the identification of 144 metabolites, with the most notable changes in the arginine metabolism, with a 1.4-fold increase in citrulline levels in JHU083 treated mice (Parveen et al., 2023).

A multi-tissue metabolite profiling study was conducted by using gas chromatography and mass spectrometry (GCMS) in female C57BL/6 mice (2 and 5 months old) infected with low-dose aerosol infection of *Mtb* H37Rv. It was demonstrated that distinct tissue metabolomic profiles existed between the mice of different age groups after infection, despite the lung bacillary load being similar till 6-weeks post-challenge in both the groups. In particular, a deregulated tissue-specific amino acid metabolism signature was observed in mice of different age groups, with the signature being more pronounced in the 5-months old mice. Additionally, it was proposed that the older age group mice could more efficiently control the infection due to reduced levels of mannose detected in their lungs, which is one of the sugars that is required for *Mtb* growth. The authors also suggested that targeting amino acid metabolic pathways could be helpful in developing adjunctive therapies (Pahwa et al., 2024). Considering the above-mentioned studies, it seems that metabolomics could be a potentially valuable tool in enhancing our understanding of TB disease by determining unique metabolic signatures that arise in specific conditions and also aid in the development of adjunctive host therapies against TB.

Since its inception, proteomics has gained considerable attention as it provides detailed insights into cellular processes, which cannot be apprehended by genomics or transcriptomics (Southan, 2004; Gengenbacher et al., 2014; Bisht et al., 2019). Proteomic profiling allows identification of proteins, which get altered upon specific conditions such as, after infection or upon treatment, thereby, aiding in identification of biomarkers for diagnosis, treatment or prevention of a disease (Kanabalan et al., 2021). However, due to the complexity of analyses and its sensitivity, the application of proteomics has yet to reach a translational stage for *in vivo* investigations pertaining to TB. Despite, these limitations, the variations observed between mRNA abundance (transcriptomics) and the corresponding protein levels indicate that proteomics provides a distinct and more correlated analysis that explains physiological responses to *Mtb* infection in true terms (Vogel and Marcotte, 2012; Bespyatykh et al., 2017).

A proteomics study conducted to define detailed molecular maps of human granulomas using LC-MS-MS led to the identification of ~3000 proteins and some lipids in the lungs of human TB subjects and *Mtb* infected NZW rabbits (Marakalala et al., 2016). The results suggested that different compartments within granulomas exhibit unique molecular signatures. It was observed that the center of granulomas has a proinflammatory milieu with the abundance of LTA4H and TNF-α, ROS mediators alongside antimicrobial effectors like cathelicidin along with proinflammatory eicosanoids. In contrast, the caseous center displayed an anti-inflammatory environment. Importantly, the data obtained was consistent across human and rabbit tissue samples. It was thus, proposed that balance between anti-inflammatory and proinflammatory mediators determines the outcome of infection (Marakalala et al., 2016).

Moreover, a mouse model has also been applied for validation of proteomics data generated from human samples. A study conducted using free-solution isoelectric focusing combined with high resolution LTQ Orbitrap Velos mass spectrometry to identify *Mtb* specific antigens from TB infected human lung tissues resulted in the identification of six *Mtb*-associated peptides. Out of these, a 34 amino acid peptide, PKAp (serine/threonine–protein kinase), was found to elicit *Mtb*-specific cellular responses with enhanced proliferation of CD8+ T-cell along with a strong cytotoxic lymphocyte (CTL) response. C57BL/6 female mice were immunized with PKAp peptide to validate the above results and it was observed that cellular levels of IFN-γ were increased in both the lungs and spleen without resulting in any immunopathogenesis. The results indicated that PKAp could be considered as a novel antigen, which can be used for development of vaccines (Yu et al., 2015).

To recapitulate, we believe that multi-omics can be employed to project a comprehensive landscape of a disease including differentiating between active and latent TB infections, predicting the risk of disease progression, or detecting markers that may be specifically associated with drug resistant TB infections. Hence, a system biology approach integrating global genomic, transcriptional, proteomic, and metabolomic profiles would provide a complete view of the disease and give in-depth information about the host-pathogen interactions. This would ultimately allow for more efficient diagnosis, enable the identification of novel anti-TB therapeutics, and pave the way for the development of personalized treatment options as well as host-directed therapies (Hasin et al., 2017; Goff et al., 2020; Guha et al., 2024).

## 4 Cellular models for studying tuberculosis

For many years, various animal models have been employed for studying complexities of TB infection, including granuloma formulation, host-pathogen interactions and latency. However, none of these models recapitulates the disease’s heterogeneity as it manifests in human. There has always been purpose-oriented use of *in vivo* models aimed at addressing specific scientific questions. While these models remain imperative for advancing our understanding of TB, it is important to consider the ethical issues related with the use of animals and to follow the principle of 3R (replace, reduce and refine) when conducting animal experiments (Franco and Rezzani, 2024).

In the recent years, *in vitro* models have emerged as powerful tools to investigate the interactions between the host and *Mtb*. These models often provide valuable insights that are challenging to achieve with animal models, further strengthening our research efforts in this critical area.

### 4.1 Cell lines and two-dimensional *in vitro* models

Given the crucial role of macrophages during *Mtb* infection, numerous studies have focused on *in vitro* models of infection to understand the pathogen’s behavior within the host. These include primary macrophages, immortalized cell lines and induced pluripotent stem cells (Keiser and Purdy, 2017). These models have been instrumental in studying the various strategies adapted by *Mtb* to survive within the host, such as phagosome maturation arrest, modulation of host cell death pathways, resistance to anti-mycobacterial compounds, alterations in the host signaling pathways and granuloma formation (Jordao et al., 2008; Keiser and Purdy, 2017).

Immortalized murine macrophage cell lines, such as RAW264.7 and J774 as well as primary cells like BMDMs, are predominant choice for *Mtb* infection studies. Both RAW264.7 and J774 are adherent macrophage cells derived from BALB/c mice, which can produce various TB associated cytokines, including TNF-α, IL-6 and IFN-*β* as well as release nitric oxide (NO) in response to bacterial infection and various other stimuli (Raschke et al., 1978; Stuehr and Marletta, 1987; Adams et al., 1993; Perez et al., 2000; Indrigo et al., 2003; Rao et al., 2005; Rao et al., 2006). It is well-established that NO produced by nitric oxide synthase 2 (NOS2), is critical host defense mechanism in controlling TB infection (Gross et al., 2014).

Primary macrophages derived from C57BL/6 mice and various knockout mice strains have been useful for studying the role of specific proteins and pathways involved in innate immunity. For example, primary cells isolated from mice deficient in NADPH oxidase and NOS2 were useful for studying the role of various detoxifying systems in mycobacteria (Bogdan, 2015). *Mtb* encodes several detoxifying genes, such as *sodA*, *katG*, NADH-dependent peroxidase, *aphC*, *aphD*, *dlaT* and *lpd* and mutations in these genes were shown to severely impair bacterial growth (Dussurget et al., 2001; Edwards et al., 2001; Ng et al., 2004; Shi and Ehrt, 2006). Additionally, several studies in macrophages have elucidated the role of *Mtb* cell wall lipids and components of ESX-1 secretion system in phagosome maturation arrest (Vergne et al., 2004; Pethe et al., 2004; MacGurn and Cox, 2007).

In addition to murine models, human monocytic cell lines, such as U937 and THP-1 are widely used for studying *Mtb* biology *in vitro*. Amongst these, human monocytic leukemia cell line, THP-1 is most extensively studied due to its ease of culturing, yielding unlimited number of cells for conducting different experiments. Differentiation of THP-1 cells into macrophages can be stimulated via phorbol 12-myristate 13-acetate (PMA), after which they become adherent, exhibit lysozyme activity, express macrophage-specific surface markers and increase their phagocytosis ability (Tsuchiya et al., 1982; Schwende et al., 1996; Daigneault et al., 2010). It is well-documented that *Mtb* induces the differentiation of macrophages to foamy macrophages *in vivo*. Thus, *in vitro* differentiation of THP-1 into foamy macrophages would serve as an excellent tool to investigate host-pathogen interactions and *Mtb* pathogenesis (Peyron et al., 2008). Several protocols are available for converting THP-1 cells into foamy macrophages by incubating them under hypoxic conditions or exposing the cell culture to fatty acids, lipoproteins or surfactant lipids (Deng et al., 2023). In addition, dormancy has also been studied in lipid loaded THP-1-derived macrophages by incubating cells under hypoxic conditions. It was observed that within these macrophages, bacteria lose their acid-fastness and become phenotypically resistant to anti-TB drugs, features that are considered indicative of a dormant state of the pathogen (Daniel et al., 2011).

Alternatively, macrophages can be extracted from healthy human donors as peripheral blood monocyte cells (PBMCs), which can be differentiated into macrophages by using granulocyte-macrophage colony-stimulating factor (GM-CSF) or macrophage colony-stimulating factor (M-CSF) or human serum (Portevin et al., 2011; Vogt and Nathan, 2011). Although, the properties of these differentiated cells are different from tissue resident cells, their use is cost-effective due to the ease of access to PBMCs. These macrophages respond to *Mtb* infection by releasing immune mediators such as TNF, IL-6 and IL-10 (Wang et al., 2010). While most studies of macrophage-pathogen interactions are focused at single cell level, these intracellular assays do not fully represent the heterogeneous microenvironment of granuloma formation (Yuan and Sampson, 2018). As such, several researchers have utilized PBMCs to develop *in vitro* 2D model to study human mycobacterial granulomas.

One such model, developed in study by Puissegur et al. demonstrated the formation of *in vitro* granulomas with morphological features and differentiation pattern similar to those of natural granulomas. They observed progressive macrophage recruitment around mycobacterial antigen- coated artificial beads or live bacteria, which further differentiated into multinucleated giant cells and epithelioid macrophages fused with surrounding macrophages and lymphocytes. This model enhanced our understanding of cell differentiation and cellular recruitment, and it could serve as a foundation for evaluating the granuloma-inducing ability of newer vaccine candidates compared to BCG (Puissegur et al., 2004).

To further understand the potential mechanisms of early granuloma formation and establishment, Guirado et al. developed an *in vitro* granuloma model from PBMCs and autologous serum. Their work showed that granuloma formation in patients with latent TB infection (LTBI) differed from those in naive individuals, with significant alterations in bacterial growth, cytokine production and lipid body accumulation. Additionally, the study highlighted unique bacterial transcriptional signatures in LTBI individuals, wherein *Mtb* exhibited a metabolic shift towards increased expression of genes involved in TCA cycle, fatty acid degradation, glyoxylate shunt and gluconeogenesis. This model greatly enhanced our fundamentals about the pathophysiology of human TB granulomas, facilitating the identification of new potential biomarkers (Guirado et al., 2015). However, this model fails to address the factors that influence the kinetics and stability of granuloma, such as the absence of fibroblasts and extracellular matrix (ECM) components. In addition, *in vitro* granuloma models have been utilized for evaluating the activity of anti-tubercular compounds in high-content screening set ups. The study showed significant differences in the minimum inhibitory concentration (MIC) of compounds against the extracellular bacteria versus bacteria within granulomas (Silva-Miranda et al., 2015).

Despite their extensive use in screening potential anti-mycobacterial molecules and investigating *Mtb* infection dynamics and immune responses, most of these *in vitro* studies rely on monolayers of cell cultured on flat surfaces. Thus, these models lack physiological microenvironment and fail to mimic the tissue architecture, receptor topography and gradients of oxygen, nutrients, and metabolites as well as the three-dimensional (3D) interactions between different cell types found *in vivo*. Furthermore, these models exhibit altered morphology, gene expression and have limited ability to replicate the complex immune responses such as interactions between macrophages, dendritic cells and T cells that occur *in vivo* (Franco and Rezzani, 2024). Consequently, the recent years have witnessed a significant focus on the development of 3D *in vitro* models that can better replicate the *in vivo* tissue microenvironment. These models offer the opportunity to study complex cellular interactions and provides a more physiologically relevant tissue microenvironment for studying immune responses and cell behavior under various stress conditions.

### 4.2 3D *in vitro* granuloma models

Several studies have explained 3D *in vitro* model of *Mtb* granulomas, in which infected primary human cells were co-cultured with various matrices (Bhavanam et al., 2016). Seitzer and Gerdes reported the first 3D granuloma model by infecting PBMCs seeded in agarose-coated well with *Mtb* H37Rv or *M. bovis* at different multiplicities of infection (MOIs). They observed the formation of cell aggregates at MOI of only 1:50 (bacteria/cells) after 4 days of incubation, while higher MOI resulted in small aggregates with higher number of dead cells. Their model displayed many phenotypic features representative of granulomas, such as aggregation of primary monocytes, B-cells, T-cells, presence of macrophages, multinucleated giant cells and necrotic areas (Seitzer and Gerdes, 2003).

To study this process further, Birkness et al. combined blood lymphocytes, autologous macrophages and *Mtb* in ultra-low attachment tissue culture plates, resulting in the formation of small, round aggregates. To mimic the natural infection process, they added non-adherent PBMCs on day 2 and day 5 post-infection and observed small aggregates of CD68+ epithelioid macrophages and CD3+ lymphocytes, similar to what is observed in clinical specimens (Birkness et al., 2007). Immunological analysis of the supernatant from these infected cells revealed the presence of several cytokines involved in human granuloma formation, including IL-6, IL-8, IFN-γ and TNF*α*. Additionally, they found that the addition of these cytokines significantly enhance the formation of aggregates. These studies provide valuable tools for studying immunological changes during bacterial infection and granuloma formation. However, these models did not provide insight into the establishment of dormancy inside granulomas and reactivation of disease (Birkness et al., 2007).

Further, Kapoor et al. employed a different approach to develop an *in vitro* model of TB granuloma by culturing human PBMCs in a collagen matrix with a low-dose of *Mtb* to study dormancy and resuscitation upon immune suppression. This model demonstrated several features similar to *in vivo* human TB granulomas, such as formation of multinucleated giant cells, reduction in CD4+ T-cell counts and secretion of various cytokines and chemokines. *Mtb* within these granulomas displayed several characteristics of dormancy, including, loss of acid-fastness, lipid bodies accumulation, tolerance to rifampicin and changes in the gene expression profile. Notably, they observed reactivation of dormant *Mtb* upon immune suppression by using anti-TNF*α* monoclonal antibodies (Kapoor et al., 2013). Despite its strength in studying latency, this model is relatively low throughput and faces limitation in studying dynamics over time due to technical challenges. Additionally, the requirement for collagenase for removal of the cells from the ECM for downstream processing also restricts its utility (Elkington et al., 2019). Recently, a study by Berry et al. introduced a novel microscale *in vitro* granuloma platform to study the signaling of soluble factors between granuloma and its surrounding microenvironment following infection. Using an open microfluidic stacks platform, they cultured *M. bovis* BCG infected monocyte-derived macrophages that could be integrated with different microenvironment cues through spatial and temporal stacking. This system resulted in generation of 3D cell aggregates encapsulating *Mtb* that secreted increased levels of proinflammatory cytokines, such as IL-6, VEGF and TNF*α*. They further extended their study by co-culturing human vascular endothelial cells with *Mtb*-infected macrophages to understand the role of granuloma associated angiogenesis (Berry et al., 2020). Additionally, several other studies have employed 3D *in vitro* granuloma model to study other mycobacterial diseases caused by other *M. leprae, M. bovis* and *M. avium subsp. paratuberculosis* (Puissegur et al., 2004; Birkness et al., 2007; Kapoor et al., 2013; Wang et al., 2013).

### 4.3 Multicellular lung tissue model

The studies described above do not fully account for the diverse cell types that are present in a *Mtb* infected lung tissue. To address this gap, researchers have developed *in vitro* human lung tissue models to study early granuloma formation (BéruBé et al., 2011; Hoang et al., 2012; Parasa et al., 2014; Braian et al., 2015; Parasa et al., 2017). The development of first human lung tissue model to study granuloma formation was reported by Parasa et al., who introduced *Mtb*-infected macrophages into an established *in vitro* lung tissue model. They observed the clustering of macrophages at the site of infection, suggesting the formation of early TB granuloma. This study also demonstrated that ESAT-6, component of ESX-1 secretion system, is required for early granuloma formation as *Mtb* mutant strain lacking RD1 region or ESAT-6 was unable to induce clustering of macrophages (Parasa et al., 2014). This finding is consistent with previous *in vivo* studies providing a unique platform to study host-pathogen interactions for the development of new therapeutic strategies (Davis and Ramakrishnan, 2009; Parasa et al., 2014).

In addition to this, researchers have investigated the role of host matrix metalloproteases (MMPs) in the formation of granuloma and bacterial growth within *Mtb* infected tissue. Human lung-derived cells and primary human monocyte-derived macrophages were utilized to model tissue lung model, which showed upregulation of several MMPs, including MMP-1, MMP-3, MMP-9 and MMP-12. Further, the use of marimastat, a global MMP inhibitor, in *Mtb*-infected lung tissue model resulted in reduction of mycobacterial growth and granuloma formation. A drawback to this study, however, is the use of global MMP inhibitor, which does not provide information into the specific role of individual MMPs in granuloma formation. Similar results regarding the involvement of MMPs at the site of necrosis and tissue damage have been observed in several animal models and in human lung tissue biopsies from patients with TB (Parasa et al., 2017).

Another notable study by Braian et al. showed the development of 3D lung model consisting of human lung-specific epithelial cells, *Mtb*-infected primary macrophages or monocytes seeded onto a matrix of collagen embedded fibroblasts prepared on a transwell filter. Exposure of the culture to air resulted in the stratification of epithelial cells and secretion of mucus at the apical surface. This model was particularly useful for 3D visualization of the entire lung tissue and for studying the migration of immune cells in the tissue during granuloma formation.

However, these studies have some limitations that include their inability to translate into high throughput platforms and the absence of other immune cells such as neutrophils and lymphocytes, which are typically present in a granuloma structures. Addressing these limitations could further strengthen the use of these models in understanding intricacies of disease.

### 4.4 Bioelectrospray 3D model

Workman et al. employed the bioelectrospray method to produce customizable microspheres containing extracellular matrix (ECM) components and THP-1 cells. Cell encapsulation within microsphere was achieved using a biocompatible cross-linking polymer, alginate, in a calcium chloride gelling bath. This system offers the advantage of easy downstream analysis as cells can be quickly released from the spheres using EDTA or sodium citrate. The researchers investigated the effect of various parameters such as voltage, flow rate and nozzle size on the size and stability of the microspheres. They compared the size of spheres generated with or without collagen addition to alginate, highlighting the role of ECM in host-pathogen interaction (Workman et al., 2014).

Subsequently, in another study it was demonstrated that *Mtb* has lower proliferation rate in collagen-containing microspheres (Tezera et al., 2017). The group also observed increased apoptosis, altered energy balance and secretion of various proinflammatory cytokines (IL-1β, TNF-α, IFN-γ, IL-6, IL-8 and MCP-1) in the presence of collagen, all of which favors host ability to control infection. The study further investigated several emerging therapeutic interventions, including the effects of cytokine supplementation on microspheres, host-directed therapy through PGE2 augmentation and immunoaugmentation with ESAT-6 and CFP-10 specific T cell lines. These findings align with several *in vivo* studies, positioning this system to serve as powerful tool for discerning the protective and pathological immune responses (Tezera et al., 2017). Additionally, bioelectrospray 3D model of TB granuloma formation has been used to investigate the mechanisms of immunopathology in TB, by specifically examining the role of MMP inhibitor in matrix degradation (Walker et al., 2017).

Apart from this, bioengineering approach have been employed to compare the sensitivity of *Mtb* to PZA in standard culture conditions versus within the 3D microspheres. Notably, the researchers observed upregulation of multiple stress-related mycobacterial genes at day 14 as well as rapid killing of bacteria by PZA inside these microspheres (Bielecka et al., 2017). This system is highly versatile, wherein cell numbers can be modulated along with changes in the composition of ECM, size of sphere, challenge dose and the surrounding media, making it a promising tool for addressing various human infections. One of the major advantages of this microsphere system is its integration potential with microfluidic platform, enabling the study of antibiotic response modulation over time to replicate the drug pharmacokinetics observed in patients during treatment. In their preliminary screening, they developed a microfluidic system with two inlets and one exit channel, facilitating smooth flow of medium through wells containing encapsulated microspheres. They demonstrated dose-dependent killing of *Mtb* with a stepwise increase in rifampicin concentration, which was consistent with clinical findings in patients (Barrila et al., 2010; Pasipanodya et al., 2013; Bielecka et al., 2017).

Like other models, this system also has some limitations, especially, the lack of vasculature, and incorporation of other immune cells once cells are embedded within the microspheres. Furthermore, the anatomical constraints that occur during infection are lost due to absence of spatial organisation seen in the lung, which makes it difficult to fully replicate complexity of granuloma. Hence, to better mimic these features, modifications such as dual encapsulation system are needed to replicate multiple microenvironments such as the caseous central core and hypoxic conditions, in order to more effectively study TB immunopathogenesis (Elkington et al., 2019; Franco and Rezzani, 2024).

### 4.5 Organoids

Advances in the stem cell biology have created an exciting opportunity to grow human tissues in dishes that closely resemble organs *in vitro*. Organoids are self-organizing, multicellular 3D aggregates that mimic structure, function, cellular heterogeneity and behavior of human tissues *in vitro* (Iakobachvili and Peters, 2017). These organoids can be derived from either adult stem cells (ASCs), embryonic stem cells, induced pluripotent stem cells (iPSCs) or tissue biopsies. Stem cells have the capacity for self-renewal and the source of these stem cells have profound impact on the types of cells present in the resulting organoids. For instance, organoids derived from ASCs are generally polarized, cystic structures consisting mostly of epithelial cells, whereas organoids derived from iPSCs tend to be more complex, containing both epithelial and non-epithelial cell types (Dichtl et al., 2024; Franco and Rezzani, 2024). Additionally, lung organoids derived from adult tissue biopsies or bronchoalveolar lavage fluid include a wide range of cells such as basal, club, goblet and ciliated cells. Organoid technology has proven useful in studying in infectious diseases, enabling researchers to better understand the host responses, pathogen survival and cell tropism (Mills and Estes, 2016; Heo et al., 2018; Hui et al., 2018; Sachs et al., 2019; Bagayoko et al., 2021).

Human airway organoids (AOs) have been used to investigate the very early steps of mycobacterial infection, particularly those caused by *Mtb* and *M. abscessus* (Mabs). Researchers have observed differences in the replication potential, cytokines secretion profile and antimicrobial peptides between these pathogens inside AOs. While the growth of *Mtb* was controlled, Mabs readily replicated more freely inside the lumen of AOs, highlighting the hospitable environment of the airways for non-tuberculous mycobacteria (NTM). Further, they attempted to co-culture human monocyte-derived macrophages with *M. bovis* BCG infected organoids and observed the movement of macrophages within the matrix towards the bacteria-containing organoids. However, this model does not fully mimic the *in vivo* environment, as macrophages were not able to traverse the basal side of hAOs to reach lumen for clearing the bacteria (Iakobachvili et al., 2022).

Recently, a group had developed a 3D human lung organoids (hLOs) model derived from human pluripotent stem cells (hPSCs). These organoids exhibited a hollow lumen structure similar to that of alveolar sacs. The model was used to study *Mtb* infection in lung epithelial cells and human macrophages by microinjecting fluorescently labelled bacteria and macrophages into the lumen of hLOs. A key advantage of this model is its ability to maintain the 3D structure and sustain the bacterial populations even after multiple passages. In addition, they have evaluated the inhibitory effects of known anti-TB drugs, rifampicin and bedaquiline, and observed a reduction in *Mtb* H37Rv growth in hLOs at each passage. This model has been utilized to explore host-directed therapies by knocking down the host genes (such as MFN2 and HERPUD1), which resulted in a significant reduction in inflammatory cytokines and intracellular *Mtb* growth, consistent with pervious findings (Lee et al., 2019; Son et al., 2023; Kim et al., 2024).

Despite the promising potential of lung organoids in TB research, there are some challenges that need to be addressed before they can be used more systematically. For example, to study host-pathogen interactions more efficiently in a lung-like environment, it is essential to incorporate macrophages and other cell types that can replicate granuloma formation. Furthermore, introduction of vasculature is necessary to create more dynamic microenvironment, allowing for better control of experimental conditions. Currently, lung organoids lack an air-liquid-mucosa interface due to their closed, cystic structure. This limitation can be overcome by using alternative microfluidic cell culture platform, such as the Lung- on-a-chip technology (Nadkarni et al., 2016; Fonseca et al., 2017).

### 4.6 Lung-on-chip

The integration of microfluidic and micro-fabricated biosystems with innovations in biological approaches has led to the development of organ-on-chip (OoC) platforms. These systems are engineered to replicate complex physiological processes of human organs that allow more accurate and ethical alternatives to animal models. Various OoC devices have been developed to model human tissues, such as the lung, intestine, heart, kidney, liver, blood vessels and blood-brain barrier (Low et al., 2021; Bhatia and Ingber, 2014). In general, each OoC platform has some defining features, including 3D microarchitecture, the integration of multiple cell types and the incorporation of relevant biomechanical forces that mimic the specific tissue environment (Shrestha et al., 2020).

One of the earliest and most notable OoC is the “Lung-on-chip” model, developed by Huh and colleagues. This model is designed to mimic the 3D structure, microenvironment and physiological processes of human lungs, including breathing movements. The system is constructed using a soft lithography-based micro-fabrication technology to create 3D micro-channel in which human alveolar epithelial cells and pulmonary micro-vascular endothelial cells were are seeded on the opposite side of culture chambers. These cells are separated by micro-porous elastomeric membrane, which mimics the alveolar-capillary interface. Subsequently, vacuum suction was applied to induce mechanical stretching of the adherent cell layers, simulating the physiological breathing motions. This proof-of-concept biomimetic system has been instrumental for conducting nano-toxicology studies and modelling complex human disease processes (Huh et al., 2010; Huh et al., 2012; Huh, 2015).

In addition to this, a human lung “small airway-on-a-chip” model has been developed by Benam et al., to study respiratory disorders, such as asthma and chronic obstructive pulmonary disease (COPD). This system includes a differentiated, mucociliary bronchial epithelium in the upper layer and an underlying micro-vascular endothelium. Fluid flow is applied to the endothelial layer, mimicking blood circulation. This model offers a powerful tool for studying both human pathophysiology and evaluating the efficacy of drugs, that can complement the preclinical research in animal models (Benam et al., 2016; Bielecka and Elkington, 2018).

Recent advancements in the field include a “Lung-on-chip” model of *Mtb* infection developed by Thacker et al. This model utilizes time-lapse microscopy to study dynamics of *Mtb* infection in alveolar epithelial cells and macrophages at an air-liquid interface, allowing for high-resolution spatiotemporal analysis. The study also examined the role of pulmonary surfactants, molecules produced by lungs to normal functioning and to respond to TB infection. The researchers found that *Mtb* grew slowly in the lung and immune cells in the presence of surfactant, indicating the protective role of surfactants in TB. This may be due to role of surfactants in pulling out proteins and fats from the surface of *Mtb*, hindering the bacteria’s ability to infect host cells. The study further indicated that pulmonary surfactant replacement formulations may serve as host-directed therapies to enhance the immune responses against TB (Thacker et al., 2020).

These advancements in lung-on-chip technology offer significant potential for TB research, particularly in the development of novel therapeutic approaches. However, further exploration is needed to address the technical hurdles and complexities associated with the disease. Refinements in these models would not only improve our understanding of respiratory diseases but also lay the groundwork for more effective drug discovery and personalized therapeutics in the future.

## 5 Concluding remarks

The capacity of an individual to clear TB infection involves a well-coordinated innate and adaptive immune response systems and thus, it is important to understand the immune response and heterogeneity associated with the disease. This review describes various *in vitro* and *in vivo* model systems to provide a comprehensive overview of the different aspects of TB research including drug discovery, vaccine development and host-pathogen interactions. Animal models remain at forefront in providing a wealth of information about host genes associated with susceptibility to TB, the immunopathogenesis of *Mtb* infection, various immune cells and responses and identification of crucial bacterial virulence determinants.

The murine model has played a significant role in TB research in identification of various important susceptibility loci to TB infections. Infact, the advent of genetic engineering and molecular techniques has allowed investigators to develop knockout and transgenic mice, leading to the identification of key immune components required for TB immunity. Moreover, most of the new TB drugs are evaluated for their therapeutic efficacy in mouse model of tuberculosis. Guinea pigs have underpinned the TB vaccine development program, providing the initial platform to identify potential vaccine candidates. Many different kinds of vaccine regimens based on recombinant BCG vaccine, auxotrophic mutants, subunit vaccines, DNA and viral vectored vaccines have been evaluated in guinea pig model of tuberculosis to establish the preliminary proof of their efficacy. NHP model of tuberculosis is an expensive system but is the closest representative to human TB. Thus, most of the promising vaccines are evaluated for their protective efficacy and immunogenicity in this model before the vaccine candidate is allowed to progress to clinical trials.

The use of animal systems have been extended to the recent omics-based technologies. With the advancements in next-generation sequencing techniques and mass spectrometry, various biomarkers and host gene signatures that differentiate between different clinical forms of TB or predict the prognosis of the disease and the treatment outcome can now be identified. Further, the review also describes the *in vitro* 2D and 3D models that have been employed to understand granuloma formation, cell surface expression of markers and the secretion of various cytokines and chemokines associated with TB. Thus, the conjugation of advanced cellular models with other “omics” approaches and *in vivo* models will offer new insights into the host-pathogen interactions, establishing a strong foundation for the development of novel therapeutic and vaccination strategies.

## References

[B1] Abel B. Thieblemont N. Quesniaux V. J. Brown N. Mpagi J. Miyake K.. (2002). Toll-like receptor 4 expression is required to control chronic Mycobacterium tuberculosis infection in mice. J. Immunol. 169, 3155–3162. doi: 10.4049/jimmunol.169.6.3155 12218133

[B2] Acharya B. Acharya A. Gautam S. Ghimire S. P. Mishra G. Parajuli N.. (2020). Advances in diagnosis of Tuberculosis: an update into molecular diagnosis of Mycobacterium tuberculosis. Mol. Biol. Rep. 47, 4065–4075. doi: 10.1007/s11033-020-05413-7 32248381

[B3] Adams L. B. Fukutomi Y. Krahenbuhl J. L. (1993). Regulation of murine macrophage effector functions by lipoarabinomannan from mycobacterial strains with different degrees of virulence. Infect. Immun. 61, 4173–4181. doi: 10.1128/iai.61.10.4173-4181.1993 8406806 PMC281141

[B4] Agarwal P. Gordon S. Martinez F. O. (2021). Foam cell macrophages in tuberculosis. Front. Immunol. 12, 775326. doi: 10.3389/fimmu.2021.775326 34975863 PMC8714672

[B5] Ahamad N. Gupta S. Parashar D. (2022). Using omics to study leprosy, tuberculosis, and other mycobacterial diseases. Front. Cell. Infect. Microbiol. 12, 792617. doi: 10.3389/fcimb.2022.792617 35281437 PMC8908319

[B6] Ai J. W. Ruan Q. L. Liu Q. H. Zhang W. H. (2016). Updates on the risk factors for latent tuberculosis reactivation and their managements. Emerg. microb. infect. 5, 1–8. doi: 10.1038/emi.2016.10 PMC4777925 26839146

[B7] Aiello A. Najafi-Fard S. Goletti D. (2023). Initial immune response after exposure to Mycobacterium tuberculosis or to SARS-COV-2: similarities and differences. Front. Immunol. 14, 1244556. doi: 10.3389/fimmu.2023.1244556 37662901 PMC10470049

[B8] Alsayed S. S. Gunosewoyo H. (2023). Tuberculosis: pathogenesis, current treatment regimens and new drug targets. Int. J. Mol. Sci. 24, 5202. doi: 10.3390/ijms24065202 36982277 PMC10049048

[B9] (2024). WHO Global TB report. Available online at: https://www.who.int/teams/global-tuberculosis-programme/tb-reports/global-tuberculosis-report-2024 (Accessed February 5, 2025).

[B10] Bafica A. Scanga C. A. Feng C. G. Leifer C. Cheever A. Sher A. (2005). TLR9 regulates Th1 responses and cooperates with TLR2 in mediating optimal resistance to Mycobacterium tuberculosis. J. Exp. Med. 202, 1715–1724. doi: 10.1084/jem.20051782 16365150 PMC2212963

[B11] Bagayoko S. Leon-Icaza S. A. Pinilla M. Hessel A. Santoni K. Péricat D.. (2021). Host phospholipid peroxidation fuels ExoU-dependent cell necrosis and supports Pseudomonas aeruginosa-driven pathology. PloS Pathog. 17, e1009927. doi: 10.1371/journal.ppat.1009927 34516571 PMC8460005

[B12] Banasik B. N. Perry C. L. Keith C. A. Bourne N. Schäfer H. Milligan G. N. (2019). Development of an anti-Guinea pig CD4 monoclonal antibody for depletion of CD4+ T cells *in vivo* . J. immunol. Methods 474, 112654. doi: 10.1016/j.jim.2019.112654 31421081 PMC6829055

[B13] Baram P. Soltysik L. Condoulis W. (1971). The *in vitro* assay of tuberculin hypersensitivity in Macaca mulatta sensitized with Bacille Calmette Guerin cell wall vaccine and/or infected with virulent Mycobacterium tuberculosis. Lab. Anim. Sci. 21 (5), 727–733. doi: 10.1159/000460011 4329237

[B14] Barclay W. R. Anacker R. L. Brehmer W. Leif W. Ribi E. (1970). Aerosol-induced tuberculosis in subhuman primates and the course of the disease after intravenous BCG vaccination. Infect. Immun. 2, 574–582. doi: 10.1128/iai.2.5.574-582.1970 16557880 PMC416053

[B15] Barrila J. Radtke A. L. Crabbé A. Sarker S. F. Herbst-Kralovetz M. M. Ott C. M.. (2010). Organotypic 3D cell culture models: using the rotating wall vessel to study host–pathogen interactions. Nat. Rev. Microbiol. 8, 791–801. doi: 10.1038/nrmicro2423 20948552

[B16] Basaraba R. J. Dailey D. D. McFarland C. T. Shanley C. A. Smith E. E. McMurray D. N.. (2006). Lymphadenitis as a major element of disease in the Guinea pig model of tuberculosis. Tuberculosis 86, 386–394. doi: 10.1016/j.tube.2005.11.003 16473044

[B17] Beamer G. L. Turner J. (2005). Murine models of susceptibility to tuberculosis. Archivum Immunol. Et Therapiae Experimentalis-English Edit. 53, 469. 16407780

[B18] Behring E. V. (1890). Ueber das Zustandekommen der Diphtherie-Immunität und der Tetanus-Immunität bei Thieren. Dtsch. Med. Wschr. 16, 1145–1147. doi: 10.1055/s-0029-1207609 5843503

[B19] Bellamy R. Ruwende C. Corrah T. McAdam K. P. Whittle H. C. Hill A. V. (1998). Variations in the NRAMP1 gene and susceptibility to tuberculosis in West Africans. New Engl. J. Med. 338, 640–644. doi: 10.1056/NEJM199803053381002 9486992

[B20] Benam K. H. Villenave R. Lucchesi C. Varone A. Hubeau C. Lee H. H.. (2016). Small airway-on-a-chip enables analysis of human lung inflammation and drug responses *in vitro* . Nat. Methods 13, 151–157. doi: 10.1038/nmeth.3697 26689262

[B21] Berg I. A. Filatova L. V. Ivanovsky R. N. (2002). Inhibition of acetate and propionate assimilation by itaconate via propionyl-CoA carboxylase in isocitrate lyase-negative purple bacterium Rhodospirillum rubrum. FEMS Microbiol. Lett. 216, 49–54. doi: 10.1111/j.1574-6968.2002.tb11413.x 12423751

[B22] Berry S. B. Gower M. S. Su X. Seshadri C. Theberge A. B. (2020). A modular microscale granuloma model for immune-microenvironment signaling studies *in vitro* . Front. bioeng. Biotechnol. 8, 931. doi: 10.3389/fbioe.2020.00931 32974300 PMC7461927

[B23] BéruBé K. Gibson C. Job C. Prytherch Z. (2011). Human lung tissue engineering: a critical tool for safer medicines. Cell Tissue bank. 12, 11–13. doi: 10.1007/s10561-010-9204-6 20824355

[B24] Bespyatykh J. A. Shitikov E. A. Ilina E. N. (2017). Proteomics for the investigation of mycobacteria. Acta Naturae (англоязычная версия) 9, 15–25. doi: 10.32607/20758251-2017-9-1-15-25 PMC5406656 28461970

[B25] Betts J. C. Lukey P. T. Robb L. C. McAdam R. A. Duncan K. (2002). Evaluation of a nutrient starvation model of Mycobacterium tuberculosis persistence by gene and protein expression profiling. Mol. Microbiol. 43 (3), 717–731. doi: 10.1046/j.1365-2958.2002.02779.x 11929527

[B26] Bhatia S. N. Ingber D. E. (2014). Microfluidic organs-on-chips. Nat. Biotechnol. 32, 760–772. doi: 10.1038/nbt.2989 25093883

[B27] Bhavanam S. Rayat G. R. Keelan M. Kunimoto D. Drews S. J. (2016). Understanding the pathophysiology of the human TB lung granuloma using *in vitro* granuloma models. Future Microbiol. 11, 1073–1089. doi: 10.2217/fmb-2016-0005 27501829

[B28] Bielecka M. K. Elkington P. (2018). Advanced cellular systems to study tuberculosis treatment. Curr. Opin. Pharmacol. 42, 16–21. doi: 10.1016/j.coph.2018.06.005 29990957

[B29] Bielecka M. K. Tezera L. B. Zmijan R. Drobniewski F. Zhang X. Jayasinghe S.. (2017). A bioengineered three-dimensional cell culture platform integrated with microfluidics to address antimicrobial resistance in tuberculosis. MBio 8, 10–1128. doi: 10.1128/mbio.02073-16 PMC5296599 28174307

[B30] Birkness K. A. Guarner J. Sable S. B. Tripp R. A. Kellar K. L. Bartlett J.. (2007). An *in vitro* model of the leukocyte interactions associated with granuloma formation in Mycobacterium tuberculosis infection. Immunol. Cell Biol. 85, 160–168. doi: 10.1038/sj.icb.7100019 17199112

[B31] Bisht D. Sharma D. Sharma D. Singh R. Gupta V. K. (2019). Recent insights into Mycobacterium tuberculosis through proteomics and implications for the clinic. Expert Rev. Proteomics 16, 443–456. doi: 10.1080/14789450.2019.1608185 31032653

[B32] Bogdan C. (2015). Nitric oxide synthase in innate and adaptive immunity: an update. Trends Immunol. 36, 161–178. doi: 10.1016/j.it.2015.01.003 25687683

[B33] Boom W. H. Schaible U. E. Achkar J. M. (2021). The knowns and unknowns of latent Mycobacterium tuberculosis infection. J. Clin. Invest. 131 (3), e136222. doi: 10.1172/JCI136222 33529162 PMC7843221

[B34] Borah K. Xu Y. McFadden J. (2021). Dissecting host-pathogen interactions in TB using systems-based omic approaches. Front. Immunol. 12, 762315. doi: 10.3389/fimmu.2021.762315 34795672 PMC8593131

[B35] Bradley D. J. (1977). Regulation of Leishmania populations within the host. II. genetic control of acute susceptibility of mice to Leishmania donovani infection. Clin. Exp. Immunol. 30, 130. 606434 PMC1541182

[B36] Braian C. Svensson M. Brighenti S. Lerm M. Parasa V. R. (2015). A 3D human lung tissue model for functional studies on Mycobacterium tuberculosis infection. J. Visual. Exper.: JoVE 104), 53084. doi: 10.3791/53084 PMC4692636 26485646

[B37] Brandt L. Skeiky Y. A. Alderson M. R. Lobet Y. Dalemans W. Turner O. C.. (2004). The protective effect of the Mycobacterium bovis BCG vaccine is increased by coadministration with the Mycobacterium tuberculosis 72-kilodalton fusion polyprotein Mtb72F in M. tuberculosis-infected Guinea pigs. Infect. Immun. 72, 6622–6632. doi: 10.1128/IAI.72.11.6622-6632.2004 15501795 PMC523007

[B38] Bucsan A. N. Mehra S. Khader S. A. Kaushal D. (2019). The current state of animal models and genomic approaches towards identifying and validating molecular determinants of Mycobacterium tuberculosis infection and tuberculosis disease. Pathog. Dis. 77, ftz037. doi: 10.1093/femspd/ftz037 31381766 PMC6687098

[B39] Burel J. G. Babor M. Pomaznoy M. Lindestam Arlehamn C. S. Khan N. Sette A.. (2019). Host transcriptomics as a tool to identify diagnostic and mechanistic immune signatures of tuberculosis. Front. Immunol. 10, 221. doi: 10.3389/fimmu.2019.00221 30837989 PMC6389658

[B40] Cappelli G. Volpe E. Grassi M. Liseo B. Colizzi V. Mariani F. (2006). Profiling of Mycobacterium tuberculosis gene expression during human macrophage infection: upregulation of the alternative sigma factor G, a group of transcriptional regulators, and proteins with unknown function. Res. Microbiol. 157, 445–455. doi: 10.1016/j.resmic.2005.10.007 16483748

[B41] Capuano S. V. III Croix D. A. Pawar S. Zinovik A. Myers A. Lin P. L.. (2003). Experimental Mycobacterium tuberculosis infection of cynomolgus macaques closely resembles the various manifestations of human M. tuberculosis infection. Infect. Immun. 71, 5831–5844. doi: 10.1128/IAI.71.10.5831-5844.2003 14500505 PMC201048

[B42] Caruso A. M. Serbina N. Klein E. Triebold K. Bloom B. R. Flynn J. L. (1999). Mice deficient in CD4 T cells have only transiently diminished levels of IFN-γ, yet succumb to tuberculosis. J. Immunol. 162, 5407–5416. doi: 10.4049/jimmunol.162.9.5407 10228018

[B43] Cervino A. C. L. Lakiss S. Sow O. Hill A. V. S. (2000). Allelic association between the NRAMP1 gene and susceptibility to tuberculosis in Guinea–Conakry. Ann. Hum. Genet. 64, 507–512. doi: 10.1046/j.1469-1809.2000.6460507.x 11281214

[B44] Chai Q. Wang L. Liu C. H. Ge B. (2020). New insights into the evasion of host innate immunity by Mycobacterium tuberculosis. Cell. Mol. Immunol. 17, 901–913. doi: 10.1038/s41423-020-0502-z 32728204 PMC7608469

[B45] Chandra P. Grigsby S. J. Philips J. A. (2022). Immune evasion and provocation by Mycobacterium tuberculosis. Nat. Rev. Microbiol. 20, 750–766. doi: 10.1038/s41579-022-00763-4 35879556 PMC9310001

[B46] Chang S. Y. Chen M. L. Lee M. R. Liang Y. C. Lu T. P. Wang J. Y.. (2018). SP110 polymorphisms are genetic markers for vulnerability to latent and active tuberculosis infection in Taiwan. Dis. Markers 2018 (1), 4687380. doi: 10.1155/2018/4687380 30627224 PMC6304864

[B47] Chaudhary K. Darling J. A. Fohl L. M. Sullivan W. J. Donald R. G. Pfefferkorn E. R.. (2004). Purine salvage pathways in the apicomplexan parasite Toxoplasma gondii. J. Biol. Chem. 279, 31221–31227. doi: 10.1074/jbc.M404232200 15140885

[B48] Claman H. N. (1972). Corticosteroids and lymphoid cells. New Engl. J. Med. 287, 388–397. doi: 10.1056/NEJM197208242870806 5043524

[B49] Clark S. Hall Y. Williams A. (2015). Animal models of tuberculosis: Guinea pigs. Cold Spring Harbor Perspect. Med. 5, a018572. doi: 10.1101/cshperspect.a018572 PMC4448592 25524720

[B50] Comstock G. W. Livesay V. T. Woolpert S. F. (1974). The prognosis of a positive tuberculin reaction in childhood and adolescence. Am. J. Epidemiol. 99, 131–138. doi: 10.1093/oxfordjournals.aje.a121593 4810628

[B51] Converse P. J. Dannenberg A. M. Estep J. E. Sugisaki K. Abe Y. Schofield B. H.. (1996). Cavitary tuberculosis produced in rabbits by aerosolized virulent tubercle bacilli. Infect. Immun. 64, 4776–4787. doi: 10.1128/iai.64.11.4776-4787.1996 8890239 PMC174445

[B52] Cooper A. M. Dalton D. K. Stewart T. A. Griffin J. P. Russell D. G. Orme I. M. (1993). Disseminated tuberculosis in interferon gamma gene-disrupted mice. J. Exp. Med. 178, 2243–2247. doi: 10.1084/jem.178.6.2243 8245795 PMC2191280

[B53] Cooper A. M. Kipnis A. Turner J. Magram J. Ferrante J. Orme I. M. (2002). Mice lacking bioactive IL-12 can generate protective, antigen-specific cellular responses to mycobacterial infection only if the IL-12 p40 subunit is present. J Immunol. 168 (3), 1322–1327. doi: 10.4049/jimmunol.168.3.1322 11801672

[B54] Daigneault M. Preston J. A. Marriott H. M. Whyte M. K. Dockrell D. H. (2010). The identification of markers of macrophage differentiation in PMA-stimulated THP-1 cells and monocyte-derived macrophages. PloS One 5, e8668. doi: 10.1371/journal.pone.0008668 20084270 PMC2800192

[B55] Dakubo G. D. Dakubo G. D. (2010). The Warburg phenomenon and other metabolic alterations of cancer cells. Mitochondr. Genet. Cancer 1, 39–66. doi: 10.1007/978-3-642-11416-8

[B56] Daniel J. Maamar H. Deb C. Sirakova T. D. Kolattukudy P. E. (2011). Mycobacterium tuberculosis uses host triacylglycerol to accumulate lipid droplets and acquires a dormancy-like phenotype in lipid-loaded macrophages. PloS Pathog. 7, e1002093. doi: 10.1371/journal.ppat.1002093 21731490 PMC3121879

[B57] Daniel J. Oh T. J. Lee C. M. Kolattukudy P. E. (2007). AccD6, a member of the Fas II locus, is a functional carboxyltransferase subunit of the acyl-coenzyme A carboxylase in Mycobacterium tuberculosis. J. bacteriol. 189, 911–917. doi: 10.1128/JB.01019-06 17114269 PMC1797314

[B58] Dannenberg A. M. Jr. (2001). Pathogenesis of pulmonary Mycobacterium bovis infection: basic principles established by the rabbit model. Tuberculosis 81, 87–96. doi: 10.1054/tube.2000.0260 11463228

[B59] Dannenberg A. M. Jr. (2009). Liquefaction and cavity formation in pulmonary TB: a simple method in rabbit skin to test inhibitors. Tuberculosis 89, 243–247. doi: 10.1016/j.tube.2009.05.006 19559651

[B60] Datta M. Via L. E. Kamoun W. S. Liu C. Chen W. Seano G.. (2015). Anti-vascular endothelial growth factor treatment normalizes tuberculosis granuloma vasculature and improves small molecule delivery. Proc. Natl. Acad. Sci. 112, 1827–1832. doi: 10.1073/pnas.1424563112 25624495 PMC4330784

[B61] Davis J. M. Ramakrishnan L. (2009). The role of the granuloma in expansion and dissemination of early tuberculous infection. Cell 136, 37–49. doi: 10.1016/j.cell.2008.11.014 19135887 PMC3134310

[B62] Deb C. Lee C. M. Dubey V. S. Daniel J. Abomoelak B. Sirakova T. D.. (2009). A novel *in vitro* multiple-stress dormancy model for Mycobacterium tuberculosis generates a lipid-loaded, drug-tolerant, dormant pathogen. PloS One 4, e6077. doi: 10.1371/journal.pone.0006077 19562030 PMC2698117

[B63] Deng L. Kersten S. Stienstra R. (2023). Triacylglycerol uptake and handling by macrophages: From fatty acids to lipoproteins. Prog. Lipid Res. 92, 101250. doi: 10.1016/j.plipres.2023.101250 37619883

[B64] Dichtl S. Posch W. Wilflingseder D. (2024). The breathtaking world of human respiratory *in vitro* models: Investigating lung diseases and infections in 3D models, organoids, and lung-on-chip. Eur. J. Immunol. 54, 2250356. doi: 10.1002/eji.202250356 38361030

[B65] Diedrich C. R. Mattila J. T. Klein E. Janssen C. Phuah J. Sturgeon T. J.. (2010). Reactivation of latent tuberculosis in cynomolgus macaques infected with SIV is associated with early peripheral T cell depletion and not virus load. PloS One 5, e9611. doi: 10.1371/journal.pone.0009611 20224771 PMC2835744

[B66] Divangahi M. Behar S. M. Remold H. (2013). Dying to live: how the death modality of the infected macrophage affects immunity to tuberculosis. Adv Exp Med Biol. 783, 103–120. doi: 10.1007/978-1-4614-6111-1 23468106 PMC4678885

[B67] Divangahi M. Mostowy S. Coulombe F. Kozak R. Guillot L. Veyrier F.. (2008). NOD2-deficient mice have impaired resistance to Mycobacterium tuberculosis infection through defective innate and adaptive immunity. J. Immunol. 181, 7157–7165. doi: 10.4049/jimmunol.181.10.7157 18981137

[B68] Dorman S. E. Hatem C. L. Tyagi S. Aird K. Lopez-Molina J. Pitt M. L. M.. (2004). Susceptibility to tuberculosis: clues from studies with inbred and outbred New Zealand White rabbits. Infect. Immun. 72, 1700–1705. doi: 10.1128/IAI.72.3.1700-1705.2004 14977978 PMC356026

[B69] Driver E. R. Ryan G. J. Hoff D. R. Irwin S. M. Basaraba R. J. Kramnik I.. (2012). Evaluation of a mouse model of necrotic granuloma formation using C3HeB/FeJ mice for testing of drugs against Mycobacterium tuberculosis. Antimicrob. Agents chemother. 56, 3181–3195. doi: 10.1128/AAC.00217-12 22470120 PMC3370740

[B70] Dussurget O. Stewart G. Neyrolles O. Pescher P. Young D. Marchal G. (2001). Role of Mycobacterium tuberculosis copper-zinc superoxide dismutase. Infect. Immun. 69, 529–533. doi: 10.1128/IAI.69.1.529-533.2001 11119546 PMC97912

[B71] Dutta N. K. Mehra S. Martinez A. N. Alvarez X. Renner N. A. Morici L. A.. (2012). The stress-response factor SigH modulates the interaction between Mycobacterium tuberculosis and host phagocytes. PloS One 7, e28958. doi: 10.1371/journal.pone.0028958 22235255 PMC3250399

[B72] Edwards K. M. Cynamon M. H. Voladri R. K. Hager C. C. DeStefano M. S. Tham K. T.. (2001). Iron-cofactored superoxide dismutase inhibits host responses to Mycobacterium tuberculosis. Am. J. respiratory Crit. Care Med. 164, 2213–2219. doi: 10.1164/ajrccm.164.12.2106093 11751190

[B73] Elkington P. Lerm M. Kapoor N. Mahon R. Pienaar E. Huh D.. (2019). *In vitro* granuloma models of tuberculosis: potential and challenges. J. Infect. Dis. 219, 1858–1866. doi: 10.1093/infdis/jiz020 30929010 PMC6534193

[B74] Eruslanov E. B. Lyadova I. V. Kondratieva T. K. Majorov K. B. Scheglov I. V. Orlova M. O.. (2005). Neutrophil responses to Mycobacterium tuberculosis infection in genetically susceptible and resistant mice. Infect. Immun. 73, 1744–1753. doi: 10.1128/IAI.73.3.1744-1753.2005 15731075 PMC1064912

[B75] Esteves P. J. Abrantes J. Baldauf H. M. BenMohamed L. Chen Y. Christensen N.. (2018). The wide utility of rabbits as models of human diseases. Exp. Mol. Med. 50, 1–10. doi: 10.1038/s12276-018-0094-1 PMC5964082 29789565

[B76] Eum S. Y. Kong J. H. Hong M. S. Lee Y. J. Kim J. H. Hwang S. H.. (2010). Neutrophils are the predominant infected phagocytic cells in the airways of patients with active pulmonary TB. Chest 137, 122–128. doi: 10.1378/chest.09-0903 19749004 PMC2803122

[B77] Fernández-García M. Rey-Stolle F. Boccard J. Reddy V. P. García A. Cumming B. M.. (2020). Comprehensive examination of the mouse lung metabolome following Mycobacterium tuberculosis infection using a multiplatform mass spectrometry approach. J. Proteome Res. 19, 2053–2070. doi: 10.1021/acs.jproteome.9b00868 32285670 PMC7199213

[B78] Filipe-Santos O. Bustamante J. Chapgier A. Vogt G. de Beaucoudrey L. Feinberg J.. (2006). Inborn errors of IL-12/23-and IFN-γ-mediated immunity: molecular, cellular, and clinical features. Semin. Immunol. 18, 347–361. doi: 10.1016/j.smim.2006.07.010 16997570

[B79] Flynn J. L. Chan J. Triebold K. J. Dalton D. K. Stewart T. A. Bloom B. R. (1993). An essential role for interferon gamma in resistance to Mycobacterium tuberculosis infection. J. Exp. Med. 178, 2249–2254. doi: 10.1084/jem.178.6.2249 7504064 PMC2191274

[B80] Flynn J. L. Goldstein M. M. Chan J. Triebold K. J. Pfeffer K. Lowenstein C. J.. (1995). Tumor necrosis factor-α is required in the protective immune response against Mycobacterium tuberculosis in mice. Immunity 2, 561–572. doi: 10.1016/1074-7613(95)90001-2 7540941

[B81] Flynn J. L. Tsenova L. Izzo A. Kaplan G. (2008). “Experimental animal models of tuberculosis,” in Kaufmann S. H.E. van Helden P. Rubin E. Britton W. J.. Handbook of tuberculosis (Hoboken, NJ: Wiley-Blackwell) 2, 389–426.

[B82] Fonseca K. L. Rodrigues P. N. Olsson I. A. S. Saraiva M. (2017). Experimental study of tuberculosis: From animal models to complex cell systems and organoids. PloS Pathog. 13, e1006421. doi: 10.1371/journal.ppat.1006421 28817682 PMC5560521

[B83] Fontán P. Aris V. Ghanny S. Soteropoulos P. Smith I. (2008). Global transcriptional profile of Mycobacterium tuberculosis during THP-1 human macrophage infection. Infect. Immun. 76 (2), 717–725. doi: 10.1128/IAI.00974-07 18070897 PMC2223452

[B84] Franco C. Rezzani R. (2024). Methods and models for studying mycobacterium tuberculosis in respiratory infections. Int. J. Mol. Sci. 26, 18. doi: 10.3390/ijms26010018 39795880 PMC11719571

[B85] Frediani J. K. Jones D. P. Tukvadze N. Uppal K. Sanikidze E. Kipiani M.. (2014). Plasma metabolomics in human pulmonary tuberculosis disease: a pilot study. PloS One 9, e108854. doi: 10.1371/journal.pone.0108854 25329995 PMC4198093

[B86] Gaber T. Strehl C. Buttgereit F. (2017). Metabolic regulation of inflammation. Nat. Rev. Rheumatol. 13, 267–279. doi: 10.1038/nrrheum.2017.37 28331208

[B87] Ganguly R. Durieux M. F. Waldman R. H. (1976). Macrophage function in vitamin C-deficient Guinea pigs. Am. J. Clin. Nutr. 29, 762–765. doi: 10.1093/ajcn/29.7.762 937230

[B88] Gao P. S. Fujishima S. Mao X. Q. Remus N. Kanda M. Enomoto T.. (2000). Genetic variants of NRAMP1 and active tuberculosis in Japanese populations. Clin. Genet. 58 (1), 74–6. doi: 10.1034/j.1399-0004.2000.580113.x 10945666

[B89] Gautam U. S. Foreman T. W. Bucsan A. N. Veatch A. V. Alvarez X. Adekambi T.. (2018). *In vivo* inhibition of tryptophan catabolism reorganizes the tuberculoma and augments immune-mediated control of Mycobacterium tuberculosis. Proc. Natl. Acad. Sci. 115, E62–E71. doi: 10.1073/pnas.1711373114 29255022 PMC5776797

[B90] Gell P. G. H. Benacerraf B. (1961). Studies on hypersensitivity: IV. The relationship between contact and delayed sensitivity: A study on the specificity of cellular immune reactions. J. Exp. Med. 113, 571–585. doi: 10.1084/jem.113.3.571 13704282 PMC2137369

[B91] Gengenbacher M. Duque-Correa M. A. Kaiser P. Schuerer S. Lazar D. Zedler U.. (2017). NOS2-deficient mice with hypoxic necrotizing lung lesions predict outcomes of tuberculosis chemotherapy in humans. Sci. Rep. 7, 8853. doi: 10.1038/s41598-017-09177-2 28821804 PMC5562869

[B92] Gengenbacher M. Mouritsen J. Schubert O. T. Aebersold R. Kaufmann S. H. (2014). Mycobacterium tuberculosis in the proteomics era. Mol. Genet. Mycobacteria 2, 239–260. doi: 10.1128/microbiolspec.MGM2-0020-2013 26105825

[B93] Goff A. Cantillon D. Muraro Wildner L. Waddell S. J. (2020). Multi-omics technologies applied to tuberculosis drug discovery. Appl. Sci. 10, 4629. doi: 10.3390/app10134629

[B94] Gormus B. J. Blanchard J. L. Alvarez X. H. Didier P. J. (2004). Evidence for a rhesus monkey model of asymptomatic tuberculosis. J. Med. primatol. 33, 134–145. doi: 10.1111/j.1600-0684.2004.00062.x 15102070

[B95] Gosain T. P. Chugh S. Rizvi Z. A. Chauhan N. K. Kidwai S. Thakur K. G.. (2024). Mycobacterium tuberculosis strain with deletions in menT3 and menT4 is attenuated and confers protection in mice and Guinea pigs. Nat. Commun. 15, 5467. doi: 10.1038/s41467-024-49246-5 38937463 PMC11211403

[B96] Goto Y. Buschman E. Skamene E. (1989). Regulation of host resistance to Mycobacterium intracellulare *in vivo* and *in vitro* by the Bcg gene. Immunogenetics. 30 (3), 218–221. doi: 10.1007/BF02421210 2673999

[B97] Gros P. Skamene E. Forget A. (1981). Genetic control of natural resistance to Mycobacterium bovis (BCG) in mice. J. Immunol. (Baltimore Md.: 1950) 127, 2417–2421. doi: 10.4049/jimmunol.127.6.2417 6795274

[B98] Gross T. J. Kremens K. Powers L. S. Brink B. Knutson T. Domann F. E.. (2014). Epigenetic silencing of the human NOS2 gene: rethinking the role of nitric oxide in human macrophage inflammatory responses. J. Immunol. 192, 2326–2338. doi: 10.4049/jimmunol.1301758 24477906 PMC3943971

[B99] Grover A. Taylor J. Troudt J. Keyser A. Arnett K. Izzo L.. (2009). Kinetics of the immune response profile in Guinea pigs after vaccination with Mycobacterium bovis BCG and infection with Mycobacterium tuberculosis. Infect. Immun. 77, 4837–4846. doi: 10.1128/IAI.00704-09 19737892 PMC2772515

[B100] Guha P. Dutta S. Murti K. Charan J. K. Pandey K. Ravichandiran V.. (2024). The integration of omics: A promising approach to personalized tuberculosis treatment. Med. Omics 12, 100033. doi: 10.1016/j.meomic.2024.100033

[B101] Guirado E. Mbawuike U. Keiser T. L. Arcos J. Azad A. K. Wang S. H.. (2015). Characterization of host and microbial determinants in individuals with latent tuberculosis infection using a human granuloma model. MBio 6, 10–1128. doi: 10.1128/mBio.02537-14 PMC4337582 25691598

[B102] Gupta U. D. Katoch V. M. (2005). Animal models of tuberculosis. Tuberculosis 85, 277–293. doi: 10.1016/j.tube.2005.08.008 16249122

[B103] Harper J. Skerry C. Davis S. L. Tasneen R. Weir M. Kramnik I.. (2012). Mouse model of necrotic tuberculosis granulomas develops hypoxic lesions. J. Infect. Dis. 205, 595–602. doi: 10.1093/infdis/jir786 22198962 PMC3266133

[B104] Harth G. Horwitz M. A. (2003). Inhibition of Mycobacterium tuberculosis glutamine synthetase as a novel antibiotic strategy against tuberculosis: demonstration of efficacy in *vivo* . Infect. Immun. 71, 456–464. doi: 10.1128/IAI.71.1.456-464.2003 12496196 PMC143262

[B105] Harth G. Masleša-Galić S. Tullius M. V. Horwitz M. A. (2005). All four Mycobacterium tuberculosis glnA genes encode glutamine synthetase activities but only GlnA1 is abundantly expressed and essential for bacterial homeostasis. Mol. Microbiol. 58, 1157–1172. doi: 10.1111/j.1365-2958.2005.04899.x 16262797

[B106] Hasin Y. Seldin M. Lusis A. (2017). Multi-omics approaches to disease. Genome Biol. 18, 1–15. doi: 10.1186/s13059-017-1215-1 28476144 PMC5418815

[B107] Heo I. Dutta D. Schaefer D. A. Iakobachvili N. Artegiani B. Sachs N.. (2018). Modelling Cryptosporidium infection in human small intestinal and lung organoids. Nat. Microbiol. 3, 814–823. doi: 10.1038/s41564-018-0177-8 29946163 PMC6027984

[B108] Heppleston A. G. (1949). Quantitative air-borne tuberculosis in the rabbit: The course of human type infection. J. Exp. Med. 89, 597. doi: 10.1084/jem.89.6.597 18129861 PMC2135897

[B109] Hillier S. Charnetzky W. (1981). Glyoxylate bypass enzymes in Yersinia species and multiple forms of isocitrate lyase in Yersinia pestis. J. bacteriol. 145, 452–458. doi: 10.1128/jb.145.1.452-458.1981 7462147 PMC217293

[B110] Hiromatsu K. Dascher C. C. LeClair K. P. Sugita M. Furlong S. T. Brenner M. B.. (2002). Induction of CD1-restricted immune responses in Guinea pigs by immunization with mycobacterial lipid antigens. J. Immunol. 169, 330–339. doi: 10.4049/jimmunol.169.1.330 12077262

[B111] Hoang A. T. N. Chen P. Juarez J. Sachamitr P. Billing B. Bosnjak L.. (2012). Dendritic cell functional properties in a three-dimensional tissue model of human lung mucosa. Am. J. Physiology-Lung Cell. Mol. Physiol. 302, L226–L237. doi: 10.1152/ajplung.00059.2011 22101763

[B112] Hoff D. R. Ryan G. J. Driver E. R. Ssemakulu C. C. De Groote M. A. Basaraba R. J.. (2011). Location of intra-and extracellular M. tuberculosis populations in lungs of mice and Guinea pigs during disease progression and after drug treatment. PloS One 6, e17550. doi: 10.1371/journal.pone.0017550 21445321 PMC3061964

[B113] Huang Z. Luo Q. Guo Y. Chen J. Xiong G. Peng Y.. (2015). Mycobacterium tuberculosis-induced polarization of human macrophage orchestrates the formation and development of tuberculous granulomas in *vitro* . PloS One 10, e0129744. doi: 10.1371/journal.pone.0129744 26091535 PMC4474964

[B114] Huh D. (2015). A human breathing lung-on-a-chip. Ann. Am. Thoracic Soc. 12, S42–S44. doi: 10.1513/AnnalsATS.201410-442MG PMC5467107 25830834

[B115] Huh D. Leslie D. C. Matthews B. D. Fraser J. P. Jurek S. Hamilton G. A.. (2012). A human disease model of drug toxicity–induced pulmonary edema in a lung-on-a-chip microdevice. Sci. Trans. Med. 4, 159ra147. doi: 10.1126/scitranslmed.3004249 PMC8265389 23136042

[B116] Huh D. Matthews B. D. Mammoto A. Montoya-Zavala M. Hsin H. Y. Ingber D. E. (2010). Reconstituting organ-level lung functions on a chip. Science 328, 1662–1668. doi: 10.1126/science.1188302 20576885 PMC8335790

[B117] Hui K. P. Ching R. H. Chan S. K. Nicholls J. M. Sachs N. Clevers H.. (2018). Tropism, replication competence, and innate immune responses of influenza virus: an analysis of human airway organoids and ex-vivo bronchus cultures. Lancet Respiratory Med. 6, 846–854. doi: 10.1016/S2213-2600(18)30236-4 30001996

[B118] Hunter L. Ruedas-Torres I. Agulló-Ros I. Rayner E. Salguero F. J. (2023). Comparative pathology of experimental pulmonary tuberculosis in animal models. Front. Vet. Sci. 10, 1264833. doi: 10.3389/fvets.2023.1264833 37901102 PMC10602689

[B119] Hyde J. E. (2007). Targeting purine and pyrimidine metabolism in human apicomplexan parasites. Curr. Drug Targets 8, 31–47. doi: 10.2174/138945007779315524 17266529 PMC2720675

[B120] Iakobachvili N. Leon-Icaza S. A. Knoops K. Sachs N. Mazères S. Simeone R.. (2022). Mycobacteria–host interactions in human bronchiolar airway organoids. Mol. Microbiol. 117, 682–692. doi: 10.1111/mmi.v117.3 34605588 PMC9298242

[B121] Iakobachvili N. Peters P. J. (2017). Humans in a dish: the potential of organoids in modeling immunity and infectious diseases. Front. Microbiol. 8, 2402. doi: 10.3389/fmicb.2017.02402 29259597 PMC5723307

[B122] Indrigo J. Hunter R. L. Jr. Actor J. K. (2003). Cord factor trehalose 6, 6′-dimycolate (TDM) mediates trafficking events during mycobacterial infection of murine macrophages. Microbiology 149, 2049–2059. doi: 10.1099/mic.0.26226-0 12904545

[B123] Irwin S. M. Gruppo V. Brooks E. Gilliland J. Scherman M. Reichlen M. J.. (2014). Limited activity of clofazimine as a single drug in a mouse model of tuberculosis exhibiting caseous necrotic granulomas. Antimicrob. Agents chemother. 58, 4026–4034. doi: 10.1128/AAC.02565-14 24798275 PMC4068578

[B124] Irwin S. M. Prideaux B. Lyon E. R. Zimmerman M. D. Brooks E. J. Schrupp C. A.. (2016). Bedaquiline and pyrazinamide treatment responses are affected by pulmonary lesion heterogeneity in Mycobacterium tuberculosis infected C3HeB/FeJ mice. ACS Infect. Dis. 2, 251–267. doi: 10.1021/acsinfecdis.5b00127 27227164 PMC4874602

[B125] Jafari M. Nasiri M. R. Sanaei R. Anoosheh S. Farnia P. Sepanjnia A.. (2016). The NRAMP1, VDR, TNF-α, ICAM1, TLR2 and TLR4 gene polymorphisms in Iranian patients with pulmonary tuberculosis: A case–control study. Infect. Genet. Evol. 39, 92–98. doi: 10.1016/j.meegid.2016.01.013 26774366

[B126] Jain R. Dey B. Dhar N. Rao V. Singh R. Gupta U. D.. (2008). Enhanced and enduring protection against tuberculosis by recombinant BCG-Ag85C and its association with modulation of cytokine profile in lung. PloS One 3, e3869. doi: 10.1371/journal.pone.0003869 19052643 PMC2586085

[B127] Jain R. Dey B. Tyagi A. K. (2012). Development of the first oligonucleotide microarray for global gene expression profiling in Guinea pigs: defining the transcription signature of infectious diseases. BMC Genomics 13, 1–11. doi: 10.1186/1471-2164-13-520 23031549 PMC3475082

[B128] Jeevan A. Yoshimura T. Lee K. E. McMurray D. N. (2003). Differential expression of gamma interferon mRNA induced by attenuated and virulent Mycobacterium tuberculosis in Guinea pig cells after Mycobacterium bovis BCG vaccination. Infect. Immun. 71, 354–364. doi: 10.1128/IAI.71.1.354-364.2003 12496185 PMC143318

[B129] Jia J. Zhang M. Cao Z. Hu X. Lei S. Zhang Y.. (2024). The rabbit model for spinal tuberculosis: An overview. J. Orthop. Surg. 32, 10225536241266703. doi: 10.1177/10225536241266703 39033332

[B130] Jilani T. N. Avula A. Zafar Gondal A. Siddiqui A. H. (2020). “Active Tuberculosis,” in StatPearls (StatPearls Publishing, Treasure Island, FL, USA). 30020618

[B131] Jordao L. Bleck C. K. Mayorga L. Griffiths G. Anes E. (2008). On the killing of mycobacteria by macrophages. Cell. Microbiol. 10, 529–548. doi: 10.1111/j.1462-5822.2007.01067.x 17986264

[B132] Kanabalan R. D. Lee L. J. Lee T. Y. Chong P. P. Hassan L. Ismail R.. (2021). Human tuberculosis and Mycobacterium tuberculosis complex: A review on genetic diversity, pathogenesis and omics approaches in host biomarkers discovery. Microbiol. Res. 246, 126674. doi: 10.1016/j.micres.2020.126674 33549960

[B133] Kaplan G. Tsenova L. (2010). “Pulmonary tuberculosis in the rabbit,” in A color atlas of comparative pathology of pulmonary tuberculosis (Boca Raton, FL), vol. 107, 130.

[B134] Kapoor N. Pawar S. Sirakova T. D. Deb C. Warren W. L. Kolattukudy P. E. (2013). Human granuloma *in vitro* model, for TB dormancy and resuscitation. PloS One 8, e53657. doi: 10.1371/journal.pone.0053657 23308269 PMC3538642

[B135] Kar R. Nangpal P. Mathur S. Singh S. Tyagi A. K. (2017). bioA mutant of Mycobacterium tuberculosis shows severe growth defect and imparts protection against tuberculosis in Guinea pigs. PloS One 12, e0179513. doi: 10.1371/journal.pone.0179513 28658275 PMC5489182

[B136] Kaufmann S. H. (2003). A short history of Robert pp.Koch’s fight against tuberculosis: those who do not remember the past are condemned to repeat it. Tuberculosis (Edinb). 83, 86–90. doi: 10.1016/s1472-9792(02)00064-1 12758195

[B137] Kawahara M. Nakasone T. Honda M. (2002). Dynamics of gamma interferon, interleukin-12 (IL-12), IL-10, and transforming growth factor β mRNA expression in primary Mycobacterium bovis BCG infection in Guinea pigs measured by a real-time fluorogenic reverse transcription-PCR assay. Infect. Immun. 70, 6614–6620. doi: 10.1128/IAI.70.12.6614-6620.2002 12438333 PMC132987

[B138] Keiser T. L. Purdy G. E. (2017). Killing Mycobacterium tuberculosis *in vitro*: what model systems can teach us. Microbiol. Spectr. 5, 10–1128. doi: 10.1128/microbiolspec.TBTB2-0028-2016 PMC6714986 28597814

[B139] Khader S. A. Guglani L. Rangel-Moreno J. Gopal R. Fallert Junecko B. A. Fountain J. J.. (2011). IL-23 is required for long-term control of Mycobacterium tuberculosis and B cell follicle formation in the infected lung. J. Immunol. 187, 5402–5407. doi: 10.4049/jimmunol.1101377 22003199 PMC3208087

[B140] Khan M. M. Ernst O. Manes N. P. Oyler B. L. Fraser I. D. Goodlett D. R.. (2019). Multi-omics strategies uncover host–pathogen interactions. ACS Infect. Dis. 5, 493–505. doi: 10.1021/acsinfecdis.9b00080 30857388 PMC13488112

[B141] Khare G. Reddy P. V. Sidhwani P. Tyagi A. K. (2013). KefB inhibits phagosomal acidification but its role is unrelated to M. tuberculosis survival in host. Sci. Rep. 3, 3527. doi: 10.1038/srep03527 24346161 PMC3866608

[B142] Kim S. Y. Choi J. A. Choi S. Kim K. K. Song C. H. Kim E. M. (2024). Advances in an *in vitro* tuberculosis infection model using human lung organoids for host-directed therapies. PloS Pathog. 20, e1012295. doi: 10.1371/journal.ppat.1012295 39052544 PMC11271890

[B143] Kjellsson M. C. Via L. E. Goh A. Weiner D. Low K. M. Kern S.. (2012). Pharmacokinetic evaluation of the penetration of antituberculosis agents in rabbit pulmonary lesions. Antimicrob. Agents chemother. 56, 446–457. doi: 10.1128/AAC.05208-11 21986820 PMC3256032

[B144] Koch R. (1882). Aetiologie der tuberculose. Berlin Klin Wochenschr 19, 221–230.

[B145] Köster S. Upadhyay S. Chandra P. Papavinasasundaram K. Yang G. Hassan A.. (2017). Mycobacterium tuberculosis is protected from NADPH oxidase and LC3-associated phagocytosis by the LCP protein CpsA. Proc. Natl. Acad. Sci. 114, E8711–E8720. doi: 10.1073/pnas.1707792114 28973896 PMC5642705

[B146] Kramnik I. Dietrich W. F. Demant P. Bloom B. R. (2000). Genetic control of resistance to experimental infection with virulent Mycobacterium tuberculosis. Proc. Natl. Acad. Sci. 97, 8560–8565. doi: 10.1073/pnas.150227197 10890913 PMC26987

[B147] Kraut J. A. Madias N. E. (2014). Lactic acidosis. New Engl. J. Med. 371, 2309–2319. doi: 10.1056/NEJMra1309483 25494270

[B148] Kroon E. E. Kinnear C. J. Orlova M. Fischinger S. Shin S. Boolay S.. (2020). An observational study identifying highly tuberculosis-exposed, HIV-1-positive but persistently TB, tuberculin and IGRA negative persons with M. tuberculosis specific antibodies in Cape Town, South Africa. EBioMedicine 61, 103053. doi: 10.1016/j.ebiom.2020.103053 33038764 PMC7648124

[B149] Kwan C. K. Ernst J. D. (2011). HIV and tuberculosis: a deadly human syndemic. Clin. Microbiol. Rev. 24, 351–376. doi: 10.1128/CMR.00042-10 21482729 PMC3122491

[B150] Laddy D. J. Bonavia A. Hanekom W. A. Kaushal D. Williams A. Roederer M.. (2018). Toward tuberculosis vaccine development: recommendations for nonhuman primate study design. Infect. Immun. 86, 10–1128. doi: 10.1128/IAI.00776-17 PMC5778361 29203540

[B151] Langermans J. A. Andersen P. van Soolingen D. Vervenne R. A. Frost P. A. van der Laan T.. (2001). Divergent effect of bacillus Calmette–Guerin (BCG) vaccination on Mycobacterium tuberculosis infection in highly related macaque species: Implications for primate models in tuberculosis vaccine research. Proc. Natl. Acad. Sci. 98, 11497–11502. doi: 10.1073/pnas.201404898 11562492 PMC58758

[B152] Lau S. K. Lam C. W. Curreem S. O. Lee K. C. Lau C. C. Chow W. N.. (2015). Identification of specific metabolites in culture supernatant of Mycobacterium tuberculosis using metabolomics: exploration of potential biomarkers. Emerg. microb. infect. 4, 1–10. doi: 10.1038/emi.2015.6 PMC4317673 26038762

[B153] Lee J. Choi J. A. Cho S. N. Son S. H. Song C. H. (2019). Mitofusin 2-deficiency suppresses Mycobacterium tuberculosis survival in macrophages. Cells 8, 1355. doi: 10.3390/cells8111355 31671648 PMC6912353

[B154] Lenaerts A. J. Hoff D. Aly S. Ehlers S. Andries K. Cantarero L.. (2007). Location of persisting mycobacteria in a Guinea pig model of tuberculosis revealed by r207910. Antimicrob. Agents chemother. 51, 3338–3345. doi: 10.1128/AAC.00276-07 17517834 PMC2043239

[B155] Leone R. D. Zhao L. Englert J. M. Sun I. M. Oh M. H. Sun I. H.. (2019). Glutamine blockade induces divergent metabolic programs to overcome tumor immune evasion. Science 366, 1013–1021. doi: 10.1126/science.aav2588 31699883 PMC7023461

[B156] Lin P. L. Dietrich J. Tan E. Abalos R. M. Burgos J. Bigbee C.. (2012a). The multistage vaccine H56 boosts the effects of BCG to protect cynomolgus macaques against active tuberculosis and reactivation of latent Mycobacterium tuberculosis infection. J. Clin. Invest. 122, 303–314. doi: 10.1172/JCI46252 22133873 PMC3248283

[B157] Lin P. L. Myers A. Smith L. K. Bigbee C. Bigbee M. Fuhrman C.. (2010). Tumor necrosis factor neutralization results in disseminated disease in acute and latent Mycobacterium tuberculosis infection with normal granuloma structure in a cynomolgus macaque model. Arthritis Rheuma. 62, 340–350. doi: 10.1002/art.27271 PMC3047004 20112395

[B158] Lin P. L. Rodgers M. Smith L. K. Bigbee M. Myers A. Bigbee C.. (2009). Quantitative comparison of active and latent tuberculosis in the cynomolgus macaque model. Infect. Immun. 77, 4631–4642. doi: 10.1128/IAI.00592-09 19620341 PMC2747916

[B159] Lin P. L. Rutledge T. Green A. M. Bigbee M. Fuhrman C. Klein E.. (2012b). CD4 T cell depletion exacerbates acute Mycobacterium tuberculosis while reactivation of latent infection is dependent on severity of tissue depletion in cynomolgus macaques. AIDS Res. Hum. retroviruses 28, 1693–1702. doi: 10.1089/aid.2012.0028 22480184 PMC3505050

[B160] Liu C. H. Liu H. Ge B. (2017). Innate immunity in tuberculosis: host defense vs pathogen evasion. Cell. Mol. Immunol. 14, 963–975. doi: 10.1038/cmi.2017.88 28890547 PMC5719146

[B161] Lorenz M. C. Fink G. R. (2001). The glyoxylate cycle is required for fungal virulence. Nature 412, 83–86. doi: 10.1038/35083594 11452311

[B162] Low L. A. Mummery C. Berridge B. R. Austin C. P. Tagle D. A. (2021). Organs-on-chips: into the next decade. Nat. Rev. Drug Discov. 20, 345–361. doi: 10.1038/s41573-020-0079-3 32913334

[B163] Luies L. Du Preez I. (2020). The echo of pulmonary tuberculosis: mechanisms of clinical symptoms and other disease-induced systemic complications. Clin. Microbiol. Rev. 33, 10–1128. doi: 10.1128/CMR.00036-20 PMC7331478 32611585

[B164] Lurie M. B. (1928). The fate of human and bovine tubercle bacilli in various organs of the rabbit. J. Exp. Med. 48, 155–182. doi: 10.1084/jem.48.2.155 19869474 PMC2131451

[B165] Lurie M. B. Abramson S. Heppleston A. G. (1952). On the response of genetically resistant and susceptible rabbits to the quantitative inhalation of human type tubercle bacilli and the nature of resistance to tuberculosis. J. Exp. Med. 95, 119. doi: 10.1084/jem.95.2.119 14907965 PMC2212059

[B166] MacGurn J. A. Cox J. S. (2007). A genetic screen for Mycobacterium tuberculosis mutants defective for phagosome maturation arrest identifies components of the ESX-1 secretion system. Infect. Immun. 75, 2668–2678. doi: 10.1128/IAI.01872-06 17353284 PMC1932882

[B167] Maiello P. DiFazio R. M. Cadena A. M. Rodgers M. A. Lin P. L. Scanga C. A.. (2018). Rhesus macaques are more susceptible to progressive tuberculosis than cynomolgus macaques: a quantitative comparison. Infect. Immun. 86, 10–1128. doi: 10.1128/IAI.00505-17 PMC5778369 28947646

[B168] Malo D. Vogan K. Vidal S. Hu J. Cellier M. Schurr E.. (1994). Haplotype mapping and sequence analysis of the mouse Nramp gene predict susceptibility to infection with intracellular parasites. Genomics 23, 51–61. doi: 10.1006/geno.1994.1458 7829102

[B169] Manabe Y. C. Dannenberg A. M. Jr. Tyagi S. K. Hatem C. L. Yoder M. Woolwine S. C.. (2003). Different strains of Mycobacterium tuberculosis cause various spectrums of disease in the rabbit model of tuberculosis. Infect. Immun. 71, 6004–6011. doi: 10.1128/IAI.71.10.6004-6011.2003 14500521 PMC201108

[B170] Manabe Y. C. Kesavan A. K. Lopez-Molina J. Hatem C. L. Brooks M. Fujiwara R.. (2008). The aerosol rabbit model of TB latency, reactivation and immune reconstitution inflammatory syndrome. Tuberculosis 88, 187–196. doi: 10.1016/j.tube.2007.10.006 18068491 PMC4477206

[B171] Marakalala M. J. Raju R. M. Sharma K. Zhang Y. J. Eugenin E. A. Prideaux B.. (2016). Inflammatory signaling in human tuberculosis granulomas is spatially organized. Nat. Med. 22, 531–538. doi: 10.1038/nm.4073 27043495 PMC4860068

[B172] Marino S. Pawar S. Fuller C. L. Reinhart T. A. Flynn J. L. Kirschner D. E. (2004). Dendritic cell trafficking and antigen presentation in the human immune response to Mycobacterium tuberculosis. J. Immunol. 173, 494–506. doi: 10.4049/jimmunol.173.1.494 15210810

[B173] Martin D. R. Sibuyi N. R. Dube P. Fadaka A. O. Cloete R. Onani M.. (2021). Aptamer-based diagnostic systems for the rapid screening of TB at the point-of-care. Diagnostics 11, 1352. doi: 10.3390/diagnostics11081352 34441287 PMC8391981

[B174] Martin C. Williams A. Hernandez-Pando R. Cardona P. J. Gormley E. Bordat Y.. (2006). The live Mycobacterium tuberculosis phoP mutant strain is more attenuated than BCG and confers protective immunity against tuberculosis in mice and Guinea pigs. Vaccine 24, 3408–3419. doi: 10.1016/j.vaccine.2006.03.017 16564606

[B175] Mattila J. T. Diedrich C. R. Lin P. L. Phuah J. Flynn J. L. (2011). Simian immunodeficiency virus-induced changes in T cell cytokine responses in cynomolgus macaques with latent Mycobacterium tuberculosis infection are associated with timing of reactivation. J. Immunol. 186, 3527–3537. doi: 10.4049/jimmunol.1003773 21317393 PMC3311978

[B176] McMurray D. N. (1994). Guinea pig model of tuberculosis. Tubercul.: pathogen. protect. control, 135–147. doi: 10.1128/9781555818357

[B177] Medina E. North R. J. (1996). Evidence inconsistent with a role for the Bcg gene (Nramp1) in resistance of mice to infection with virulent Mycobacterium tuberculosis. J. Exp. Med. 183, 1045–1051. doi: 10.1084/jem.183.3.1045 8642246 PMC2192312

[B178] Medina E. North R. J. (1998). Resistance ranking of some common inbred mouse strains to Mycobacterium tuberculosis and relationship to major histocompatibility complex haplotype and Nramp1 genotype. Immunology 93, 270–274. doi: 10.1046/j.1365-2567.1998.00419.x 9616378 PMC1364188

[B179] Mehra S. Golden N. A. Dutta N. K. Midkiff C. C. Alvarez X. Doyle L. A.. (2011). Reactivation of latent tuberculosis in rhesus macaques by coinfection with simian immunodeficiency virus. J. Med. primatol. 40, 233–243. doi: 10.1111/j.1600-0684.2011.00485.x 21781131 PMC3227019

[B180] Mehra S. Golden N. A. Stuckey K. Didier P. J. Doyle L. A. Russell-Lodrigue K. E.. (2012). The Mycobacterium tuberculosis stress response factor SigH is required for bacterial burden as well as immunopathology in primate lungs. J. Infect. Dis. 205, 1203–1213. doi: 10.1093/infdis/jis102 22402035 PMC3308902

[B181] Mehra S. Pahar B. Dutta N. K. Conerly C. N. Philippi-Falkenstein K. Alvarez X.. (2010). Transcriptional reprogramming in nonhuman primate (rhesus macaque) tuberculosis granulomas. PloS One 5, e12266. doi: 10.1371/journal.pone.0012266 20824205 PMC2930844

[B182] Méndez-Samperio P. (2010). Role of interleukin-12 family cytokines in the cellular response to mycobacterial disease. Int. J. Infect. Dis. 14, e366–e371. doi: 10.1016/j.ijid.2009.06.022 19762261

[B183] Meurs H. Santing R. E. Remie R. van der Mark T. W. Westerhof F. J. Zuidhof A. B.. (2006). A Guinea pig model of acute and chronic asthma using permanently instrumented and unrestrained animals. Nat. Protoc. 1, 840–847. doi: 10.1038/nprot.2006.144 17406316

[B184] Miller E. A. Ernst J. D. (2009). Anti-TNF immunotherapy and tuberculosis reactivation: another mechanism revealed. J. Clin. Invest. 119, 1079–1082. doi: 10.1172/JCI39143 19422095 PMC2673853

[B185] Mills M. Estes M. K. (2016). Physiologically relevant human tissue models for infectious diseases. Drug Discov. Today 21, 1540–1552. doi: 10.1016/j.drudis.2016.06.020 27352632 PMC5365153

[B186] Mitsos L. M. Cardon L. R. Fortin A. Ryan L. LaCourse R. North R. J.. (2000). Genetic control of susceptibility to infection with Mycobacterium tuberculosis in mice. Genes Immun. 1, 467–477. doi: 10.1038/sj.gene.6363712 11197687

[B187] Mitsos L. M. Cardon L. R. Ryan L. LaCourse R. North R. J. Gros P. (2003). Susceptibility to tuberculosis: a locus on mouse chromosome 19 (Trl-4) regulates Mycobacterium tuberculosis replication in the lungs. Proc. Natl. Acad. Sci. 100, 6610–6615. doi: 10.1073/pnas.1031727100 12740444 PMC164495

[B188] Moreira-Teixeira L. Tabone O. Graham C. M. Singhania A. Stavropoulos E. Redford P. S.. (2020). Mouse transcriptome reveals potential signatures of protection and pathogenesis in human tuberculosis. Nat. Immunol. 21, 464–476. doi: 10.1038/s41590-020-0610-z 32205882 PMC7116040

[B189] Mukadi Y. Perriëns J. H. St Louis M. E. Brown C. Ryder R. W. Portaels F.. (1993). Spectrum of immunodeficiency in HIV-1-infected patients with pulmonary tuberculosis in Zaire. Lancet 342, 143–146. doi: 10.1016/0140-6736(93)91346-N 8101257

[B190] Nadkarni R. R. Abed S. Draper J. S. (2016). Organoids as a model system for studying human lung development and disease. Biochem. Biophys. Res. Commun. 473, 675–682. doi: 10.1016/j.bbrc.2015.12.091 26721435

[B191] Nangpal P. Bahal R. K. Tyagi A. K. (2017). Boosting with recombinant MVA expressing M. tuberculosis α-crystallin antigen augments the protection imparted by BCG against tuberculosis in Guinea pigs. Sci. Rep. 7, 17286. doi: 10.1038/s41598-017-17587-5 29230061 PMC5725557

[B192] Ndlovu H. Marakalala M. J. (2016). Granulomas and inflammation: host-directed therapies for tuberculosis. Front. Immunol. 7, 434. doi: 10.3389/fimmu.2016.00434 27822210 PMC5075764

[B193] Ng V. H. Cox J. S. Sousa A. O. MacMicking J. D. McKinney J. D. (2004). Role of KatG catalase-peroxidase in mycobacterial pathogenesis: countering the phagocyte oxidative burst. Mol. Microbiol. 52, 1291–1302. doi: 10.1111/j.1365-2958.2004.04078.x 15165233

[B194] North R. J. LaCourse R. Ryan L. Gros P. (1999). Consequence of Nramp1 deletion to Mycobacterium tuberculosis infection in mice. Infect. Immun. 67, 5811–5814. doi: 10.1128/IAI.67.11.5811-5814.1999 10531233 PMC96959

[B195] Ogus A. C. Yoldas B. Ozdemir T. Uguz A. Olcen S. Keser I.. (2004). The Arg753GLn polymorphism of the human toll-like receptor 2 gene in tuberculosis disease. Eur. Respiratory J. 23, 219–223. doi: 10.1183/09031936.03.00061703 14979495

[B196] Oh M. H. Sun I. H. Zhao L. Leone R. D. Sun I. M. Xu W.. (2020). Targeting glutamine metabolism enhances tumor-specific immunity by modulating suppressive myeloid cells. J. Clin. Invest. 130, 3865–3884. doi: 10.1172/JCI131859 32324593 PMC7324212

[B197] Okamoto Y. Umemura M. Yahagi A. O’Brien R. L. Ikuta K. Kishihara K.. (2010). Essential role of IL-17A in the formation of a mycobacterial infection-induced granuloma in the lung. J. Immunol. 184, 4414–4422. doi: 10.4049/jimmunol.0903332 20212094

[B198] Ordway D. Palanisamy G. Henao-Tamayo M. Smith E. E. Shanley C. Orme I. M.. (2007). The cellular immune response to Mycobacterium tuberculosis infection in the Guinea pig. J. Immunol. 179, 2532–2541. doi: 10.4049/jimmunol.179.4.2532 17675515

[B199] Orme I. M. Basaraba R. J. (2014). The formation of the granuloma in tuberculosis infection. Semin. Immunol. 26, 601–609. doi: 10.1016/j.smim.2014.09.009 25453231

[B200] Orme I. M. Ordway D. J. (2016). Mouse and Guinea pig models of tuberculosis. Microbiol. Spectr. 4, 10–1128. doi: 10.1128/microbiolspec.TBTB2-0002-2015 27726797

[B201] Padilla-Carlin D. J. McMurray D. N. Hickey A. J. (2008). The Guinea pig as a model of infectious diseases. Comp. Med. 58, 324–340. 18724774 PMC2706043

[B202] Pahwa F. Chaudhary S. Dayal A. Nanda R. K. (2024). Lung Mycobacterium tuberculosis infection perturbs metabolic pathways in non-pulmonary tissues. bioRxiv, 2024–2002. doi: 10.1101/2024.02.09.579656

[B203] Pan H. Yan B. S. Rojas M. Shebzukhov Y. V. Zhou H. Kobzik L.. (2005). Ipr1 gene mediates innate immunity to tuberculosis. Nature 434, 767–772. doi: 10.1038/nature03419 15815631 PMC1388092

[B204] Parasa V. R. Muvva J. R. Rose J. F. Braian C. Brighenti S. Lerm M. (2017). Inhibition of tissue matrix metalloproteinases interferes with Mycobacterium tuberculosis-induced granuloma formation and reduces bacterial load in a human lung tissue model. Front. Microbiol. 8, 2370. doi: 10.3389/fmicb.2017.02370 29259583 PMC5723394

[B205] Parasa V. R. Rahman M. J. Ngyuen Hoang A. T. Svensson M. Brighenti S. Lerm M. (2014). Modeling Mycobacterium tuberculosis early granuloma formation in experimental human lung tissue. Dis. Models Mech. 7, 281–288. doi: 10.1242/dmm.013854 PMC3917249 24203885

[B206] Park J. Kim H. Kwon K. W. Choi H. H. Kang S. M. Hong J. J.. (2020). Toll-like receptor 4 signaling-mediated responses are critically engaged in optimal host protection against highly virulent Mycobacterium tuberculosis K infection. Virulence 11, 430–445. doi: 10.1080/21505594.2020.1766401 32403973 PMC7239029

[B207] Parveen S. Shen J. Lun S. Zhao L. Alt J. Koleske B.. (2023). Glutamine metabolism inhibition has dual immunomodulatory and antibacterial activities against Mycobacterium tuberculosis. Nat. Commun. 14, 7427. doi: 10.1038/s41467-023-43304-0 37973991 PMC10654700

[B208] Pasipanodya J. G. McIlleron H. Burger A. Wash P. A. Smith P. Gumbo T. (2013). Serum drug concentrations predictive of pulmonary tuberculosis outcomes. J. Infect. Dis. 208, 1464–1473. doi: 10.1093/infdis/jit352 23901086 PMC3789573

[B209] Perez R. L. Roman J. Roser S. Little C. Olsen M. Indrigo J.. (2000). Cytokine message and protein expression during lung granuloma formation and resolution induced by the mycobacterial cord factor trehalose-6, 6′-dimycolate. J. Interfer. Cytok. Res. 20, 795–804. doi: 10.1089/10799900050151067 11032399

[B210] Pethe K. Swenson D. L. Alonso S. Anderson J. Wang C. Russell D. G. (2004). Isolation of Mycobacterium tuberculosis mutants defective in the arrest of phagosome maturation. Proc. Natl. Acad. Sci. 101, 13642–13647. doi: 10.1073/pnas.0401657101 15340136 PMC518761

[B211] Peyron P. Vaubourgeix J. Poquet Y. Levillain F. Botanch C. Bardou F.. (2008). Foamy macrophages from tuberculous patients’ granulomas constitute a nutrient-rich reservoir for M. tubercul. persist. PloS Pathog. 4, e1000204. doi: 10.1371/journal.ppat.1000204 PMC2575403 19002241

[B212] Plant J. Glynn A. A. (1976). Genetics of resistance to infection with Salmonella typhimurium in mice. J. Infect. Dis. 133, 72–78. doi: 10.1093/infdis/133.1.72 1107437

[B213] Portevin D. Gagneux S. Comas I. Young D. (2011). Human macrophage responses to clinical isolates from the Mycobacterium tuberculosis complex discriminate between ancient and modern lineages. PloS Pathog. 7, e1001307. doi: 10.1371/journal.ppat.1001307 21408618 PMC3048359

[B214] Post F. A. Wood R. Pillay G. P. (1995). Pulmonary tuberculosis in HIV infection: radiographic appearance is related to CD4+ T-lymphocyte count. Tubercle Lung Dis. 76, 518–521. doi: 10.1016/0962-8479(95)90527-8 8593372

[B215] Pradhan G. Shrivastva R. Mukhopadhyay S. (2018). Mycobacterial PknG targets the Rab7l1 signaling pathway to inhibit phagosome–lysosome fusion. J. Immunol. 201, 1421–1433. doi: 10.4049/jimmunol.1800530 30037848

[B216] Puissegur M. P. Botanch C. Duteyrat J. L. Delsol G. Caratero C. Altare F. (2004). An *in vitro* dual model of mycobacterial granulomas to investigate the molecular interactions between mycobacteria and human host cells. Cell. Microbiol. 6, 423–433. doi: 10.1111/j.1462-5822.2004.00371.x 15056213

[B217] Puri R. V. Reddy P. V. Tyagi A. K. (2013). Secreted acid phosphatase (SapM) of Mycobacterium tuberculosis is indispensable for arresting phagosomal maturation and growth of the pathogen in Guinea pig tissues. PloS One 8, e70514. doi: 10.1371/journal.pone.0070514 23923000 PMC3724783

[B218] Qualls J. E. Murray P. J. (2016). Immunometabolism within the tuberculosis granuloma: amino acids, hypoxia, and cellular respiration. Semin. immunopathol. 38, 139–152. doi: 10.1007/s00281-015-0534-0 26490974 PMC4779414

[B219] Rachman H. Strong M. Schaible U. Schuchhardt J. Hagens K. Mollenkopf H.. (2006a). Mycobacterium tuberculosis gene expression profiling within the context of protein networks. Microb. infect. 8, 747–757. doi: 10.1016/j.micinf.2005.09.011 16513384

[B220] Rachman H. Strong M. Ulrichs T. Grode L. Schuchhardt J. Mollenkopf H.. (2006b). Unique transcriptome signature of Mycobacterium tuberculosis in pulmonary tuberculosis. Infect. Immun. 74, 1233–1242. doi: 10.1128/IAI.74.2.1233-1242.2006 16428773 PMC1360294

[B221] Rais R. Jancarik A. Tenora L. Nedelcovych M. Alt J. Englert J.. (2016). Discovery of 6-diazo-5-oxo-l-norleucine (DON) prodrugs with enhanced CSF delivery in monkeys: a potential treatment for glioblastoma. J. med. Chem. 59, 8621–8633. doi: 10.1021/acs.jmedchem.6b01069 27560860

[B222] Rao V. Fujiwara N. Porcelli S. A. Glickman M. S. (2005). Mycobacterium tuberculosis controls host innate immune activation through cyclopropane modification of a glycolipid effector molecule. J. Exp. Med. 201, 535–543. doi: 10.1084/jem.20041668 15710652 PMC2213067

[B223] Rao V. Gao F. Chen B. Jacobs W. R. Glickman M. S. (2006). Trans-cyclopropanation of mycolic acids on trehalose dimycolate suppresses Mycobacterium tuberculosis–induced inflammation and virulence. J. Clin. Invest. 116, 1660–1667. doi: 10.1172/JCI27335 16741578 PMC1464906

[B224] Raschke W. C. Baird S. Ralph P. Nakoinz I. (1978). Functional macrophage cell lines transformed by Abelson leukemia virus. Cell 15, 261–267. doi: 10.1016/0092-8674(78)90101-0 212198

[B225] Ravesloot-Chávez M. M. Van Dis E. Stanley S. A. (2021). The innate immune response to Mycobacterium tuberculosis infection. Annu. Rev. Immunol. 39, 611–637. doi: 10.1146/annurev-immunol-093019-010426 33637017

[B226] Reddy P. V. Puri R. V. Chauhan P. Kar R. Rohilla A. Khera A.. (2013). Disruption of mycobactin biosynthesis leads to attenuation of Mycobacterium tuberculosis for growth and virulence. J. Infect. Dis. 208, 1255–1265. doi: 10.1093/infdis/jit250 23788726

[B227] Reddy P. V. Puri R. V. Khera A. Tyagi A. K. (2012). Iron storage proteins are essential for the survival and pathogenesis of Mycobacterium tuberculosis in THP-1 macrophages and the Guinea pig model of infection. J. bacteriol. 194, 567–575. doi: 10.1128/JB.05553-11 22101841 PMC3264086

[B228] Reed S. G. Coler R. N. Dalemans W. Tan E. V. DeLa Cruz E. C. Basaraba R. J.. (2009). Defined tuberculosis vaccine, Mtb72F/AS02A, evidence of protection in cynomolgus monkeys. Proc. Natl. Acad. Sci. 106, 2301–2306. doi: 10.1073/pnas.0712077106 19188599 PMC2650151

[B229] Reyes P. Rathod P. K. Sanchez D. J. Mrema J. E. Rieckmann K. H. Heidrich H. G. (1982). Enzymes of purine and pyrimidine metabolism from the human malaria parasite, Plasmodium falciparum. Mol. Biochem. parasitol. 5, 275–290. doi: 10.1016/0166-6851(82)90035-4 6285190

[B230] Rhoades E. R. Frank A. A. Orme I. M. (1997). Progression of chronic pulmonary tuberculosis in mice aerogenically infected with virulent Mycobacterium tuberculosis. Tubercle Lung Dis. 78, 57–66. doi: 10.1016/S0962-8479(97)90016-2 9666963

[B231] Rifat D. Prideaux B. Savic R. M. Urbanowski M. E. Parsons T. L. Luna B.. (2018). Pharmacokinetics of rifapentine and rifampin in a rabbit model of tuberculosis and correlation with clinical trial data. Sci. Trans. Med. 10, eaai7786. doi: 10.1126/scitranslmed.aai7786 PMC5969904 29618565

[B232] Routy J. P. Routy B. Graziani G. M. Mehraj V. (2016). The kynurenine pathway is a double-edged sword in immune-privileged sites and in cancer: implications for immunotherapy. Int. J. Tryptop. Res. 9, IJTR–S38355. doi: 10.4137/IJTR.S38355 PMC5063567 27773992

[B233] Rustad T. R. Harrell M. I. Liao R. Sherman D. R. (2008). The enduring hypoxic response of Mycobacterium tuberculosis. PloS One 3, e1502. doi: 10.1371/journal.pone.0001502 18231589 PMC2198943

[B234] Ryu S. Park Y. K. Bai G. H. Kim S. J. Park S. N. Kang S. (2000). 3′ UTR polymorphisms in the NRAMP1 gene are associated with susceptibility to tuberculosis in Koreans. Int. J. Tubercul. Lung Dis. 4, 577–580. 10864190

[B235] Sachs N. Papaspyropoulos A. Zomer-van-Ommen D. D. Heo I. Böttinger L. Klay D.. (2019). Long-term expanding human airway organoids for disease modeling. EMBO J. 38, e100300. doi: 10.15252/embj.2018100300 30643021 PMC6376275

[B236] Salindri A. D. Haw J. S. Amere G. A. Alese J. T. Umpierrez G. E. Magee M. J. (2021). Latent tuberculosis infection among patients with and without type-2 diabetes mellitus: results from a hospital case-control study in Atlanta. BMC Res. Notes 14, 252. doi: 10.1186/s13104-021-05662-0 34193265 PMC8247096

[B237] Sampson S. L. Dascher C. C. Sambandamurthy V. K. Russell R. G. Jacobs W. R. Jr. Bloom B. R.. (2004). Protection elicited by a double leucine and pantothenate auxotroph of Mycobacterium tuberculosis in Guinea pigs. Infect. Immun. 72, 3031–3037. doi: 10.1128/IAI.72.5.3031-3037.2004 15102816 PMC387862

[B238] Sankar P. Mishra B. B. (2023). Early innate cell interactions with Mycobacterium tuberculosis in protection and pathology of tuberculosis. Front. Immunol. 14, 1260859. doi: 10.3389/fimmu.2023.1260859 37965344 PMC10641450

[B239] Scanga C. A. Bafica A. Feng C. G. Cheever A. W. Hieny S. Sher A. (2004). MyD88-deficient mice display a profound loss in resistance to Mycobacterium tuberculosis associated with partially impaired Th1 cytokine and nitric oxide synthase 2 expression. Infect. Immun. 72, 2400–2404. doi: 10.1128/IAI.72.4.2400-2404.2004 15039368 PMC375220

[B240] Schmidt L. H. (1966). Studies on the antituberculous activity of ethambutol in monkeys. Ann. New York Acad. Sci. 135, 747–758. doi: 10.1111/j.1749-6632.1966.tb45520.x 4957084

[B241] Schnappinger D. Ehrt S. Voskuil M. I. Liu Y. Mangan J. A. Monahan I. M.. (2003). Transcriptional adaptation of Mycobacterium tuberculosis within macrophages: insights into the phagosomal environment. J. Exp. Med. 198, 693–704. doi: 10.1084/jem.20030846 12953091 PMC2194186

[B242] Schwende H. Fitzke E. Ambs P. Dieter P. (1996). Differences in the state of differentiation of THP-1 cells induced by phorbol ester and 1, 25-dihydroxyvitamin D3. J. Leuco. Biol. 59, 555–561. doi: 10.1002/jlb.59.4.555 8613704

[B243] Seitzer U. Gerdes J. (2003). Generation and characterization of multicellular heterospheroids formed by human peripheral blood mononuclear cells. Cells Tissues Organs 174, 110–116. doi: 10.1159/000071151 12835574

[B244] Serafini A. Tan L. Horswell S. Howell S. Greenwood D. J. Hunt D. M.. (2019). Mycobacterium tuberculosis requires glyoxylate shunt and reverse methylcitrate cycle for lactate and pyruvate metabolism. Mol. Microbiol. 112, 1284–1307. doi: 10.1111/mmi.v112.4 31389636 PMC6851703

[B245] Shang S. Harton M. Tamayo M. H. Shanley C. Palanisamy G. S. Caraway M.. (2011). Increased Foxp3 expression in Guinea pigs infected with W-Beijing strains of M. tuberculosis. Tuberculosis 91, 378–385. doi: 10.1016/j.tube.2011.06.001 21737349 PMC3172339

[B246] Sharma V. Sharma S. Zu Bentrup K. H. McKinney J. D. Russell D. G. Jacobs W. R.. (2000). Structure of isocitrate lyase, a persistence factor of Mycobacterium tuberculosis. Nat. Struct. Biol. 7, 663–668. doi: 10.1038/77964 10932251

[B247] Sharpe S. White A. Gleeson F. McIntyre A. Smyth D. Clark S.. (2016). Ultra low dose aerosol challenge with Mycobacterium tuberculosis leads to divergent outcomes in rhesus and cynomolgus macaques. Tuberculosis 96, 1–12. doi: 10.1016/j.tube.2015.10.004 26786648

[B248] Shi S. Ehrt S. (2006). Dihydrolipoamide acyltransferase is critical for Mycobacterium tuberculosis pathogenesis. Infect. Immun. 74, 56–63. doi: 10.1128/IAI.74.1.56-63.2006 16368957 PMC1346611

[B249] Shin J. H. Yang J. Y. Jeon B. Y. Yoon Y. J. Cho S. N. Kang Y. H.. (2011). 1H NMR-based metabolomic profiling in mice infected with Mycobacterium tuberculosis. J. Proteome Res. 10, 2238–2247. doi: 10.1021/pr101054m 21452902

[B250] Shrestha J. Razavi Bazaz S. Aboulkheyr Es H. Yaghobian Azari D. Thierry B. Ebrahimi Warkiani M.. (2020). Lung-on-a-chip: the future of respiratory disease models and pharmacological studies. Crit. Rev. Biotechnol. 40, 213–230. doi: 10.1080/07388551.2019.1710458 31906727

[B251] Sibley L. Dennis M. Sarfas C. White A. Clark S. Gleeson F.. (2016). Route of delivery to the airway influences the distribution of pulmonary disease but not the outcome of Mycobacterium tuberculosis infection in rhesus macaques. Tuberculosis 96, 141–149. doi: 10.1016/j.tube.2015.11.004 26723465

[B252] Silva-Miranda M. Ekaza E. Breiman A. Asehnoune K. Barros-Aguirre D. Pethe K.. (2015). High-content screening technology combined with a human granuloma model as a new approach to evaluate the activities of drugs against Mycobacterium tuberculosis. Antimicrob. Agents chemother. 59, 693–697. doi: 10.1128/AAC.03705-14 25348525 PMC4291390

[B253] Simmonds H. A. Duley J. A. Fairbanks L. D. McBride M. B. (1997). When to investigate for purine and pyrimidine disorders. Introduction and review of clinical and laboratory indications. J. inherit. Metab. Dis. 20, 214–226. doi: 10.1023/A:1005308923168 9211194

[B254] Simmons J. D. Stein C. M. Seshadri C. Campo M. Alter G. Fortune S.. (2018). Immunological mechanisms of human resistance to persistent Mycobacterium tuberculosis infection. Nat. Rev. Immunol. 18, 575–589. doi: 10.1038/s41577-018-0025-3 29895826 PMC6278832

[B255] Singh S. Goswami N. Tyagi A. K. Khare G. (2019). Unraveling the role of the transcriptional regulator VirS in low pH–induced responses of Mycobacterium tuberculosis and identification of VirS inhibitors. J. Biol. Chem. 294, 10055–10075. doi: 10.1074/jbc.RA118.005312 31126988 PMC6664167

[B256] Singh V. Jamwal S. Jain R. Verma P. Gokhale R. Rao K. V. (2012). Mycobacterium tuberculosis-driven targeted recalibration of macrophage lipid homeostasis promotes the foamy phenotype. Cell host Microbe 12, 669–681. doi: 10.1016/j.chom.2012.09.012 23159056

[B257] Singh R. Rao V. Shakila H. Gupta R. Khera A. Dhar N.. (2003). Disruption of mptpB impairs the ability of Mycobacterium tuberculosis to survive in Guinea pigs. Mol. Microbiol. 50, 751–762. doi: 10.1046/j.1365-2958.2003.03712.x 14617138

[B258] Smith D. W. McMurray D. N. Wiegeshaus E. H. Grover A. A. Harding G. E. (1970). Host-parasite relationships in experimental airborne tuberculosis: IV. Early events in the course of infection in vaccinated and nonvaccinated Guinea pigs. Am. Rev. Respiratory Dis. 102, 937–949. doi: 10.1164/arrd.1970.102.6.937 4991996

[B259] Soldevilla P. Vilaplana C. Cardona P. J. (2022). Mouse models for Mycobacterium tuberculosis pathogenesis: show and do not tell. Pathogens 12, 49. doi: 10.3390/pathogens12010049 36678397 PMC9865329

[B260] Solovic I. Sester M. Gomez-Reino J. J. Rieder H. L. Ehlers S. Milburn H. J.. (2010). The risk of tuberculosis related to tumour necrosis factor antagonist therapies: a TBNET consensus statement. Eur. Respir. J. (2011) 36, 1185–1206. doi: 10.1183/09031936.00028510 20530046

[B261] Somashekar B. S. Amin A. G. Rithner C. D. Troudt J. Basaraba R. Izzo A.. (2011). Metabolic profiling of lung granuloma in Mycobacterium tuberculosis infected Guinea pigs: ex vivo 1H magic angle spinning NMR studies. J. Proteome Res. 10, 4186–4195. doi: 10.1021/pr2003352 21732701

[B262] Somashekar B. S. Amin A. G. Tripathi P. MacKinnon N. Rithner C. D. Shanley C. A.. (2012). Metabolomic signatures in Guinea pigs infected with epidemic-associated W-Beijing strains of Mycobacterium tuberculosis. J. Proteome Res. 11, 4873–4884. doi: 10.1021/pr300345x 22873951

[B263] Son S. H. Lee J. Cho S. N. Choi J. A. Kim J. Nguyen T. D.. (2023). Herp regulates intracellular survival of Mycobacterium tuberculosis H37Ra in macrophages by regulating reactive oxygen species-mediated autophagy. Mbio 14, e01535–e01523. doi: 10.1128/mbio.01535-23 37800958 PMC10653826

[B264] Southan C. (2004). Has the yo-yo stopped? An assessment of human protein-coding gene number. Proteomics 4, 1712–1726. doi: 10.1002/pmic.200300700 15174140

[B265] Srivastava S. Battu M. B. Khan M. Z. Nandicoori V. K. Mukhopadhyay S. (2019). Mycobacterium tuberculosis PPE2 protein interacts with p67phox and inhibits reactive oxygen species production. J. Immunol. 203, 1218–1229. doi: 10.4049/jimmunol.1801143 31375544

[B266] Stead W. W. Senner J. W. Reddick W. T. Lofgren J. P. (1990). Racial differences in susceptibility to infection by Mycobacterium tuberculosis. N. Engl. J. Med. 322, 422–427. doi: 10.1056/NEJM199002153220702 2300105

[B267] Stewart G. R. Wernisch L. Stabler R. Mangan J. A. Hinds J. Laing K. G.. (2002). Dissection of the heat-shock response in Mycobacterium tuberculosis using mutants and microarrays. Microbiology 148, 3129–3138. doi: 10.1099/00221287-148-10-3129 12368446

[B268] Stuehr D. J. Marletta M. A. (1987). Synthesis of nitrite and nitrate in murine macrophage cell lines. Cancer Res. 47, 5590–5594. 3117354

[B269] Subbian S. Tsenova L. O’Brien P. Yang G. Koo M. S. Peixoto B.. (2011a). Phosphodiesterase-4 inhibition combined with isoniazid treatment of rabbits with pulmonary tuberculosis reduces macrophage activation and lung pathology. Am. J. Pathol. 179, 289–301. doi: 10.1016/j.ajpath.2011.03.039 21703411 PMC3123788

[B270] Subbian S. Tsenova L. O’Brien P. Yang G. Kushner N. L. Parsons S.. (2012). Spontaneous latency in a rabbit model of pulmonary tuberculosis. Am. J. Pathol. 181, pp.1711–1724. doi: 10.1016/j.ajpath.2012.07.019 PMC3483799 22960076

[B271] Subbian S. Tsenova L. Yang G. O’Brien P. Parsons S. Peixoto B.. (2011b). Chronic pulmonary cavitary tuberculosis in rabbits: a failed host immune response. Open Biol. 1, 110016. doi: 10.1098/rsob.110016 22645653 PMC3352086

[B272] Sullivan B. M. Jobe O. Lazarevic V. Vasquez K. Bronson R. Glimcher L. H.. (2005). Increased susceptibility of mice lacking T-bet to infection with Mycobacterium tuberculosis correlates with increased IL-10 and decreased IFN-γ production. J. Immunol. 175, 4593–4602. doi: 10.4049/jimmunol.175.7.4593 16177104

[B273] Sun J. Singh V. Lau A. Stokes R. W. Obregón-Henao A. Orme I. M.. (2013). Mycobacterium tuberculosis nucleoside diphosphate kinase inactivates small GTPases leading to evasion of innate immunity. PloS Pathog. 9, e1003499. doi: 10.1371/journal.ppat.1003499 23874203 PMC3715411

[B274] Tailleux L. Waddell S. J. Pelizzola M. Mortellaro A. Withers M. Tanne A.. (2008). Probing host pathogen cross-talk by transcriptional profiling of both Mycobacterium tuberculosis and infected human dendritic cells and macrophages. PloS One 3, e1403. doi: 10.1371/journal.pone.0001403 18167562 PMC2151136

[B275] Talaat A. M. Ward S. K. Wu C. W. Rondon E. Tavano C. Bannantine J. P.. (2007). Mycobacterial bacilli are metabolically active during chronic tuberculosis in murine lungs: insights from genome-wide transcriptional profiling. J. bacteriol. 189 (11), 4265–4274. doi: 10.1128/JB.00011-07 17384189 PMC1913421

[B276] Talaat A. M. Lyons R. Howard S. T. Johnston S. A. (2004). The temporal expression profile of Mycobacterium tuberculosis infection in mice. Proc. Natl. Acad. Sci. 101, 4602–4607. doi: 10.1073/pnas.0306023101 15070764 PMC384793

[B277] Tezera L. B. Bielecka M. K. Chancellor A. Reichmann M. T. Shammari B. A. Brace P.. (2017). Dissection of the host-pathogen interaction in human tuberculosis using a bioengineered 3-dimensional model. Elife 6, e21283. doi: 10.7554/eLife.21283 28063256 PMC5238961

[B278] Tezera L. B. Bielecka M. K. Ogongo P. Walker N. F. Ellis M. Garay-Baquero D. J.. (2020a). Anti-PD-1 immunotherapy leads to tuberculosis reactivation via dysregulation of TNF-α. Elife 9, e52668. doi: 10.7554/eLife.52668 32091388 PMC7058383

[B279] Tezera L. B. Mansour S. Elkington P. (2020b). Reconsidering the optimal immune response to Mycobacterium tuberculosis. Am. J. respiratory Crit. Care Med. 201, 407–413. doi: 10.1164/rccm.201908-1506PP PMC7049929 31657633

[B280] Thacker V. V. Dhar N. Sharma K. Barrile R. Karalis K. McKinney J. D. (2020). A lung-on-chip model of early Mycobacterium tuberculosis infection reveals an essential role for alveolar epithelial cells in controlling bacterial growth. Elife 9, e59961. doi: 10.7554/eLife.59961.sa2 33228849 PMC7735758

[B281] Tornheim J. A. Dooley K. E. (2017). Tuberculosis associated with HIV infection. Microbiol. Spectr. 5, 10–1128. doi: 10.1128/microbiolspec.TNMI7-0028-2016 PMC11687440 28233512

[B282] Torrado E. Cooper A. M. (2010). IL-17 and Th17 cells in tuberculosis. Cytok. Growth factor Rev. 21, 455–462. doi: 10.1016/j.cytogfr.2010.10.004 PMC3032416 21075039

[B283] Torres-García D. Cruz-Lagunas A. García-Sancho-Figueroa M. C. Fernández-Plata R. Baez-Saldaña R. Mendoza-Milla C.. (2013). Variants in toll-like receptor 9 gene influence susceptibility to tuberculosis in a Mexican population. J. Trans. Med. 11, 1–8. doi: 10.1186/1479-5876-11-220 PMC3849691 24053111

[B284] Tosh K. Campbell S. J. Fielding K. Sillah J. Bah B. Gustafson P.. (2006). Variants in the SP110 gene are associated with genetic susceptibility to tuberculosis in West Africa. Proc. Natl. Acad. Sci. 103, 10364–10368. doi: 10.1073/pnas.0603340103 16803959 PMC1502463

[B285] Tsenova L. Sokol K. Victoria H. F. Kaplan G. (1998). A combination of thalidomide plus antibiotics protects rabbits from mycobacterial meningitis-associated death. J. Infect. Dis. 177, 1563–1572. doi: 10.1086/jid.1998.177.issue-6 9607834

[B286] Tsuchiya S. Kobayashi Y. Goto Y. Okumura H. Nakae S. Konno T.. (1982). Induction of maturation in cultured human monocytic leukemia cells by a phorbol diester. Cancer Res. 42, 1530–1536. 6949641

[B287] Tullius M. V. Harth G. Horwitz M. A. (2003). Glutamine synthetase GlnA1 is essential for growth of Mycobacterium tuberculosis in human THP-1 macrophages and Guinea pigs. Infect. Immun. 71, 3927–3936. doi: 10.1128/IAI.71.7.3927-3936.2003 12819079 PMC162033

[B288] Turner O. C. Basaraba R. J. Orme I. M. (2003). Immunopathogenesis of pulmonary granulomas in the Guinea pig after infection with Mycobacterium tuberculosis. Infect. Immun. 71, 864–871. doi: 10.1128/IAI.71.2.864-871.2003 12540568 PMC145351

[B289] Turner J. Gonzalez-Juarrero M. Saunders B. M. Brooks J. V. Marietta P. Ellis D. L.. (2001). Immunological basis for reactivation of tuberculosis in mice. Infect. Immun. 69, 3264–3270. doi: 10.1128/IAI.69.5.3264-3270.2001 11292749 PMC98285

[B290] van Crevel R. Ottenhoff T. H. van der Meer J. W. (2002). Innate immunity to Mycobacterium tuberculosis. Clin. Microbiol. Rev. 15, 294–309. doi: 10.1128/CMR.15.2.294-309.2002 11932234 PMC118070

[B291] Vandal O. H. Roberts J. A. Odaira T. Schnappinger D. Nathan C. F. Ehrt S. (2009). Acid-susceptible mutants of Mycobacterium tuberculosis share hypersusceptibility to cell wall and oxidative stress and to the host environment. J. bacteriol. 191, 625–631. doi: 10.1128/JB.00932-08 19011036 PMC2620805

[B292] van den Esker M. H. Koets A. P. (2019). Application of transcriptomics to enhance early diagnostics of mycobacterial infections, with an emphasis on Mycobacterium avium ssp. paratuberculosis. Vet. Sci. 6, 59. doi: 10.3390/vetsci6030059 31247942 PMC6789504

[B293] Venketaraman V. Dayaram Y. K. Talaue M. T. Connell N. D. (2005). Glutathione and nitrosoglutathione in macrophage defense against Mycobacterium tuberculosis. Infect. Immun. 73, 1886–1889. doi: 10.1128/IAI.73.3.1886-1889.2005 15731094 PMC1064956

[B294] Vergne I. Fratti R. A. Hill P. J. Chua J. Belisle J. Deretic V. (2004). Mycobacterium tuberculosis phagosome maturation arrest: mycobacterial phosphatidylinositol analog phosphatidylinositol mannoside stimulates early endosomal fusion. Mol. Biol. Cell 15(2), 751–760. doi: 10.1091/mbc.E03-05-0307 14617817 PMC329390

[B295] Verreck F. A. Vervenne R. A. Kondova I. van Kralingen K. W. Remarque E. J. Braskamp G.. (2009). MVA. 85A boosting of BCG and an attenuated, phoP deficient M. tuberculosis vaccine both show protective efficacy against tuberculosis in rhesus macaques. PloS One 4, e5264. doi: 10.1371/journal.pone.0005264 19367339 PMC2666807

[B296] Via L. E. Lin P. L. Ray S. M. Carrillo J. Allen S. S. Eum S. Y.. (2008). Tuberculous granulomas are hypoxic in Guinea pigs, rabbits, and nonhuman primates. Infect. Immun. 76, 2333–2340. doi: 10.1128/IAI.01515-07 18347040 PMC2423064

[B297] Via L. E. Schimel D. Weiner D. M. Dartois V. Dayao E. Cai Y.. (2012). Infection dynamics and response to chemotherapy in a rabbit model of tuberculosis using [18F] 2-fluoro-deoxy-D-glucose positron emission tomography and computed tomography. Antimicrob. Agents chemother. 56, 4391–4402. doi: 10.1128/AAC.00531-12 22687508 PMC3421588

[B298] Via L. E. Weiner D. M. Schimel D. Lin P. L. Dayao E. Tankersley S. L.. (2013). Differential virulence and disease progression following Mycobacterium tuberculosis complex infection of the common marmoset (Callithrix jacchus). Infect. Immun. 81, 2909–2919. doi: 10.1128/IAI.00632-13 23716617 PMC3719573

[B299] Vidal S. M. Malo D. Vogan K. Skamene E. Gros P. (1993). Natural resistance to infection with intracellular parasites: isolation of a candidate for Bcg. Cell 73, 469–485. doi: 10.1016/0092-8674(93)90135-D 8490962

[B300] Vogel C. Marcotte E. M. (2012). Insights into the regulation of protein abundance from proteomic and transcriptomic analyses. Nat. Rev. Genet. 13, 227–232. doi: 10.1038/nrg3185 22411467 PMC3654667

[B301] Vogt G. Nathan C. (2011). *In vitro* differentiation of human macrophages with enhanced antimycobacterial activity. J. Clin. Invest. 121, 3889–3901. doi: 10.1172/JCI57235 21911939 PMC3195467

[B302] Vrieling F. Kostidis S. Spaink H. P. Haks M. C. Mayboroda O. A. Ottenhoff T. H.. (2020). Analyzing the impact of Mycobacterium tuberculosis infection on primary human macrophages by combined exploratory and targeted metabolomics. Sci. Rep. 10, 7085. doi: 10.1038/s41598-020-62911-1 32341411 PMC7184630

[B303] Walker N. F. Wilkinson K. A. Meintjes G. Tezera L. B. Goliath R. Peyper J. M.. (2017). Matrix degradation in human immunodeficiency virus type 1–Associated tuberculosis and tuberculosis immune reconstitution inflammatory syndrome: a prospective observational study. Clin. Infect. Dis. 65, 121–132. doi: 10.1093/cid/cix231 28475709 PMC5815569

[B304] Walsh G. P. Tan E. V. Dela Cruz E. C. Abalos R. M. Villahermosa L. G. Young L. J.. (1996). The Philippine cynomolgus monkey (Macaca fasicularis) provides a new nonhuman primate model of tuberculosis that resembles human disease. Nat. Med. 2, 430–436. doi: 10.1038/nm0496-430 8597953

[B305] Wang H. Maeda Y. Fukutomi Y. Makino M. (2013). An *in vitro* model of Mycobacterium leprae induced granuloma formation. BMC Infect. Dis. 13, 1–10. doi: 10.1186/1471-2334-13-279 23782413 PMC3693892

[B306] Wang C. Peyron P. Mestre O. Kaplan G. van Soolingen D. Gao Q.. (2010). Innate immune response to Mycobacterium tuberculosis Beijing and other genotypes. PloS One 5, e13594. doi: 10.1371/journal.pone.0013594 21049036 PMC2963601

[B307] Ward S. K. Abomoelak B. Marcus S. A. Talaat A. M. (2010). Transcriptional profiling of Mycobacterium tuberculosis during infection: lessons learned. Front. Microbiol. 1, 121. doi: 10.3389/fmicb.2010.00121 21738523 PMC3125582

[B308] Weljie A. M. Dowlatabadi R. Miller B. J. Vogel H. J. Jirik F. R. (2007). An inflammatory arthritis-associated metabolite biomarker pattern revealed by 1H NMR spectroscopy. J. Proteome Res. 6, 3456–3464. doi: 10.1021/pr070123j 17696462

[B309] Westermann A. J. Barquist L. Vogel J. (2017). Resolving host–pathogen interactions by dual RNA-seq. PloS Pathog. 13, e1006033. doi: 10.1371/journal.ppat.1006033 28207848 PMC5313147

[B310] White A. D. Sibley L. Sarfas C. Morrison A. Gullick J. Clark S.. (2021). MTBVAC vaccination protects rhesus macaques against aerosol challenge with M. tuberculosis and induces immune signatures analogous to those observed in clinical studies. NPJ Vacc. 6, 4. doi: 10.1038/s41541-020-00262-8 PMC7782851 33397991

[B311] WHO Tuberculosis (TB): Latent Tuberculosis Infection (LTBI)—FAQs. Available online at: https://www.who.int/tb/areas-of-work/preventive-care/ltbi/faqs/en/ (Accessed May 26 2021).

[B312] WHO End Strategy. Available online at: https://www.who.int/teams/global-tuberculosis-programme/the-end-tb-strategy.

[B313] Williams A. Hall Y. Orme I. M. (2009). Evaluation of new vaccines for tuberculosis in the Guinea pig model. Tuberculosis 89, 389–397. doi: 10.1016/j.tube.2009.08.004 19815462

[B314] Williams A. Hatch G. J. Clark S. O. Gooch K. E. Hatch K. A. Hall G. A.. (2005). Evaluation of vaccines in the EU TB Vaccine Cluster using a Guinea pig aerosol infection model of tuberculosis. Tuberculosis 85, 29–38. doi: 10.1016/j.tube.2004.09.009 15687025

[B315] Wilson G. J. Marakalala M. J. Hoving J. C. Van Laarhoven A. Drummond R. A. Kerscher B.. (2015). The C-type lectin receptor CLECSF8/CLEC4D is a key component of anti-mycobacterial immunity. Cell host Microbe 17, 252–259. doi: 10.1016/j.chom.2015.01.004 25674984 PMC4334100

[B316] Workman V. L. Tezera L. B. Elkington P. T. Jayasinghe S. N. (2014). Controlled generation of microspheres incorporating extracellular matrix fibrils for three-dimensional cell culture. Adv. Funct. mater. 24, 2648–2657. doi: 10.1002/adfm.201303891 25411575 PMC4233144

[B317] Yamada H. Udagawa T. Mizuno S. Hiramatsu K. Sugawara I. (2005). Newly designed primer sets available for evaluating various cytokines and iNOS mRNA expression in Guinea pig lung tissues by RT-PCR. Exp. Anim. 54, 163–172. doi: 10.1538/expanim.54.163 15897626

[B318] Yamamoto T. Jeevan A. Ohishi K. Nojima Y. Umemori K. Yamamoto S.. (2002). A new assay system for Guinea pig interferon biological activity. J. interfer. cytok. Res. 22, 793–797. doi: 10.1089/107999002320271387 12184917

[B319] Yan B. S. Kirby A. Shebzukhov Y. V. Daly M. J. Kramnik I. (2006). Genetic architecture of tuberculosis resistance in a mouse model of infection. Genes Immun. 7, 201–210. doi: 10.1038/sj.gene.6364288 16452998

[B320] Yan B. S. Pichugin A. V. Jobe O. Helming L. Eruslanov E. B. Gutiérrez-Pabello J. A.. (2007). Progression of pulmonary tuberculosis and efficiency of bacillus Calmette-Guerin vaccination are genetically controlled via a common sst1-mediated mechanism of innate immunity. J. Immunol. 179, 6919–6932. doi: 10.4049/jimmunol.179.10.6919 17982083

[B321] Yang H. J. Wang D. Wen X. Weiner D. M. Via L. E. (2021). One size fits all? Not in *in vivo* modeling of tuberculosis chemotherapeutics. Front. Cell. Infect. Microbiol. 11, 613149. doi: 10.3389/fcimb.2021.613149 33796474 PMC8008060

[B322] Yu Y. Jin D. Hu S. Zhang Y. Zheng X. Zheng J.. (2015). A novel tuberculosis antigen identified from human tuberculosis granulomas*. Mol. Cell. Proteomics 14, 1093–1103. doi: 10.1074/mcp.M114.045237 25605460 PMC4390254

[B323] Yue X. Zhu X. Wu L. Shi J. (2022). A comparative study of a rabbit spinal tuberculosis model constructed by local direct infection via the posterior lateral approach. Sci Rep. 12 (1), 12853. doi: 10.1038/s41598-022-16624-2 35896778 PMC9329296

[B324] Yuan T. Sampson N. S. (2018). Hit generation in TB drug discovery: from genome to granuloma. Chem. Rev. 118, 1887–1916. doi: 10.1021/acs.chemrev.7b00602 29384369 PMC5832989

[B325] Zaki H. Y. Leung K. H. Yiu W. C. Gasmelseed N. Elwali N. E. M. Yip S. P. (2012). Common polymorphisms in TLR4 gene associated with susceptibility to pulmonary tuberculosis in the Sudanese. Int. J. tubercul. Lung Dis. 16, 934–940. doi: 10.5588/ijtld.11.0517 22525209

[B326] Zhan L. Tang J. Sun M. Qin C. (2017). Animal models for tuberculosis in translational and precision medicine. Front. Microbiol. 8, 717. doi: 10.3389/fmicb.2017.00717 28522990 PMC5415616

